# Synthesis and Photophysical Properties of Tumor-Targeted Water-Soluble BODIPY Photosensitizers for Photodynamic Therapy

**DOI:** 10.3390/molecules25153340

**Published:** 2020-07-23

**Authors:** Duy Khuong Mai, Byungman Kang, Temmy Pegarro Vales, Isabel Wen Badon, Sung Cho, Joomin Lee, Eunae Kim, Ho-Joong Kim

**Affiliations:** 1 Department of Chemistry, Chosun University, Gwangju 61452, Korea; maikhuongduy@gmail.com (D.K.M.); valestemmy@gmail.com (T.P.V.); isabel.badon.isabel@gmail.com (I.W.B.); 2 Department of Chemistry, Chonnam National University, Gwangju 61186, Korea; 3 Nuclear Chemistry Research Division, Korea Atomic Energy Research Institute, 989-111 Daedeok-daero, Yuseong-gu, Daejeon 34057, Korea; alchem95@gmail.com; 4 Department of Natural Sciences, Caraga State University, Butuan City 8600, Philippines; 5 College of Food and Nutrition, Chosun University, Gwangju 61452, Korea; 6 College of Pharmacy, Chosun University, Gwangju 61452, Korea

**Keywords:** BODIPY, click reaction, lactose, photodynamic therapy, photosensitizer

## Abstract

The synthesis of three water-soluble lactose-modified 4,4-difluoro-4-bora-3a,4a-diaza-s-indacene (BODIPY)-based photosensitizers with tumor-targeting capabilities is reported, including an investigation into their photodynamic therapeutic activity on three distinct cancer cell lines (human hepatoma Huh7, cervical cancer HeLa, and breast cancer MCF-7 cell lines). The halogenated BODIPY dyes exhibited a decreased fluorescence quantum yield compared to their non-halogenated counterpart, and facilitated the efficient generation of singlet oxygen species. The synthesized dyes exhibited low cytotoxicities in the dark and high photodynamic therapeutic capabilities against the treated cancer cell lines following irradiation at 530 nm. Moreover, the incorporation of lactose moieties led to an enhanced cellular uptake of the BODIPY dyes. Collectively, the results presented herein provide promising insights for the development of photodynamic therapeutic agents for cancer treatment.

## 1. Introduction

Photodynamic therapy (PDT) is an emerging clinical strategy for local, controllable, and noninvasive cancer treatment that combines three key components: light, oxygen, and a photosensitizing (PS) agent [1,2]. The mechanism of action involves irradiation of the localized PS agents with light of appropriate wavelengths to generate cytotoxic and highly reactive oxygen species (ROS), including singlet oxygen (^1^O_2_), thereby causing tissue damage in the regions where these three key components converge [3]. Several PS agents have been reported for use in PDT, including cyclic tetrapyrroles (chlorins, porphyrins, and bacteriochlorins) and phenothiazinium-based photosensitizers [4,5,6,7]. However, the majority of these PS agents are met with numerous drawbacks confining their clinical applications, including low light-to-dark toxicity ratios, low photostability, and structural instability. In addition, these conventional PS agents are synthesized employing elaborate synthetic routes and purification processes and can only be utilized with certain solvents [8,9,10]. Hence, there is an increasing demand for the development of new classes of PDT photosensitizers that are straightforward to synthesize, highly efficient, photostable, and widely applicable under various conditions.

A promising class of fluorophore that has shown excellent potential as a PS agent over the past decade is 4,4-difluoro-4-bora-3a,4a-diaza-s-indacene (BODIPY) [7,11,12,13]. This dye has gained relevance both in fundamental research and clinical practice, primarily due to its innate advantageous photophysical characteristics, including high extinction coefficients, high fluorescence quantum yield, resistance to photobleaching [11,14], and high light-to-dark toxicity ratios, which are higher than those of conventional phenothiazinium-based PDT agents [2,5]. Apart from being easily synthesized, BODIPY dyes are amenable to several post-synthetic modifications aimed at modulating their photophysical properties and improving their ROS generation capability. In particular, the incorporation of heavy halogen atoms at the *2*,*6*-position of the BODIPY core could significantly enhance singlet-to-triplet intersystem crossing (ISC) transitions of the chromophore and consequently increase singlet ^1^O_2_ quantum yield. Such introduction of heavy atoms onto a molecule that significantly influences the rates of ISC is termed the heavy-atom effect [15]. In spite of the considerable advances in BODIPY biomarker research, many of these BODIPY derivatives have limited biological utility due to their hydrophobic nature, tendency to form aggregates in aqueous environments, and a lack of sufficient tumor selectivity [16,17,18]. Numerous synthetic approaches have been reported for enhancing the aqueous solubility and tumor-selectivity of these PS agents, including the attachment of ionizable hydrophilic groups (e.g., sulfonic acid, carboxylic acid, phosphonic acid, and ammonium groups), and biomolecules (e.g., polyethylene glycol, oligonucleotides, and carbohydrates) [19,20,21].

Among the multitude of ionizable hydrophilic moieties and biomolecules employed to improve the water solubility of BODIPY derivatives, carbohydrate groups have demonstrated a remarkable ability in balancing the biodegradability, stability, biocompatibility, and tumor-targeting efficacy of the resulting photosensitizers [22]. It is well known that carbohydrates are essential signaling molecules and play a critical role in cellular recognition events [23]. Carbohydrate-modified photosensitizers are thought to be endowed with an outstanding potential for photodynamic therapy applications due to their enhanced interactions with a number of overexpressed specific receptors in tumor cells via carbohydrate-mediated cell recognition processes [24,25]. For instance, lactose, a disaccharide comprising galactose and glucose units, has been regarded as a promising targeting ligand in cancer treatment. In an aqueous environment, the glucose moiety transforms into its chain structure, while the galactose group remains in a stable ring structure [26]. Recent developments in the field of molecular recognition have reported the targeting capabilities of the galactose and lactose-modified macromolecules against several human cancer cells, particularly hepatocellular carcinoma cells [27,28,29,30].

Despite sophisticated carbohydrate methodologies formulated by glycoscientists [31,32], the development of an effective synthetic approach that is rapid, versatile, straightforward, high-yielding, regiospecific and does not entail lengthy purification procedures remains desirable, to mitigate the ever-growing relevance in the preparation of functional carbohydrate derivatives. Owing to its aforementioned outstanding attributes, a wide array of BODIPY-carbohydrate functional materials have been prepared and have found wide applications in the fields of chemistry, biology, and material science [33,34,35]. Herein, the synthesis of a series of tumor-targeting and water soluble BODIPY-based photosensitizers conjugated with a lactose moiety via the copper iodide (CuI) catalyzed azide-alkyne cycloaddition (CuAAC) click reaction is reported. The synthesized dyes were subjected to an extensive photophysical investigation including UV/Vis absorbance and emission, fluorescence quantum yield, and singlet oxygen quantum yield measurements. In addition, the biocompatibilities and photodynamic therapeutic properties of the synthesized dyes were assessed against three distinct cancer cell lines (human hepatoma Huh7, cervical cancer HeLa, and breast cancer MCF-7 cell lines).

## 2. Results and Discussion

### 2.1. Design and Synthesis of Water-Soluble BODIPY Derivatives

**Scheme 1:**
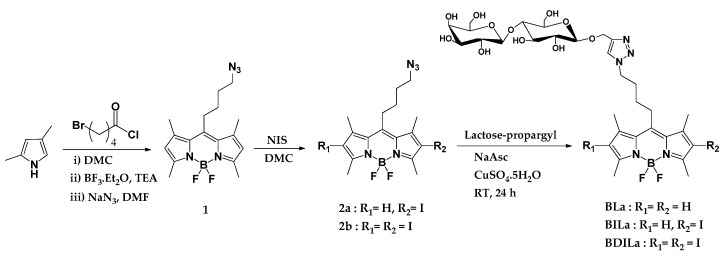
Synthesis of the tumor-targeting, lactose-modified, and water-soluble 4,4-difluoro-4-bora-3a,4a-diaza-s-indacene (BODIPY) dyes.

A three-step synthetic route of the tumor-targeting and water-soluble BODIPY derivatives is outlined in Scheme 1. The BODIPY core synthesis began with the reaction of dimethyl pyrrole with an acid chloride derivative, 5-bromovaleryl chloride, to form a dipyrromethene hydrochloride salt intermediate. This unstable intermediate subsequently complexes with the nearby boron trifluoride diethyl etherate in the presence of triethylamine, eventually producing the BODIPY core. Then, the alkyl halide moiety of BODIPY was converted in good yield to its corresponding alkyl azide (BODIPY dye 1) by treatment of the precursor dye with sodium azide (NaN_3_). The second step involves the introduction of heavy iodine atoms onto either the 2- or 2,6-position of the BODIPY core, using *N*-iodosuccinimide (NIS) as the iodine source. It has been well-established that the incorporation of such heavy atoms onto aza-BODIPY and BODIPY derivatives significantly enhances the intersystem crossing efficiency and consequently increases the singlet oxygen quantum yield via an increase in spin–orbit coupling [11,15]. BODIPY dyes 2a and 2b were afforded in excellent yields (78% and 85%, respectively) by adjusting the corresponding amounts of NIS and the reaction duration. The final step involves the attachment of the lactose moiety via the facile and straightforward CuAAC click reaction. The conjugation of the sugar substituent ultimately generated three lactose-functionalized BODIPY-based photosensitizers with intrinsic tumor-targeting and photodynamic therapeutic properties: BLa, BILa, and BDILa.

Pivotal to our synthetic approach for the preparation of the BODIPY-based PS agents was the development of a facile, simple, and economic route that would deliver reproducible results and efficient reaction yields, without employing elaborate experimental set-ups. Thus, we employed the CuAAC click reaction, which is extensively utilized for the development of a wide array of functional carbohydrate-modified BODIPY derivatives due to its efficiency, selectivity, and versatility [34]. Herein, the CuAAC click reaction was accomplished under mild reaction conditions, providing good yields and entailing simple purification techniques. The three final BODIPY dyes BLa, BILa, and BDILa were purified via a simple recrystallization method in a methanol/ether solvent system and obtained in good yields (47% to 63%). Moreover, the conjugation of the lactose moiety rendered the resulting BODIPY dyes water-soluble. The dyes BLa and BILa were completely dissolved in water, while BDILa could be readily dissolved in water by dilution from a stock solution in dimethyl sulfoxide (DMSO). Herein, the sample stock solutions (100 µM) containing 0.5% (*v*/*v*) DMSO were prepared, as commonly practiced in biology related experiments [36]. In addition, the structures of the synthesized BODIPY derivatives were confirmed by ^1^H-NMR spectroscopy, while additional ^13^C-NMR spectroscopic characterizations were performed for the final three BODIPY derivatives, BLa, BILa, and BDILa.

### 2.2. Photophysical and Theoretical Characterizations of Water-Soluble BODIPY Derivatives

**Figure 1:**
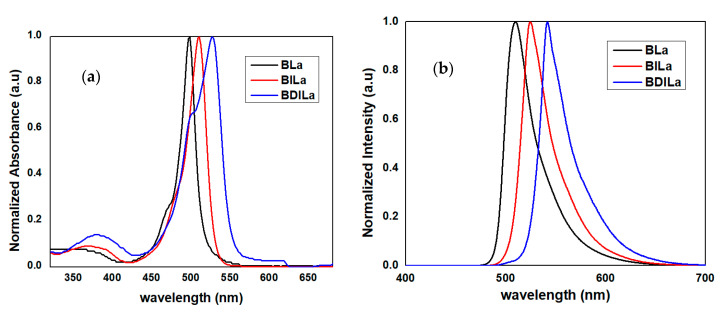
(**a**) absorption and (**b**) emission spectra of the water-soluble BODIPY-based photosensitizers BLa, BILa, and BDILa in aqueous solution. Solutions were excited at their corresponding absorption maxima.

The optical properties of the synthesized water-soluble BODIPY-based photosensitizers BLa, BILa, and BDILa were investigated through UV/Vis absorption and fluorescence spectroscopic measurements in aqueous solution containing 0.5% (*v*/*v*) DMSO. The obtained absorbance and emission spectra of the prepared BODIPY dyes are shown in Figure 1 and key values are summarized in Table 1. All of the synthesized BODIPY derivatives exhibited spectral properties characteristic of the BODIPY core. In the absorption spectra, intense absorbance bands were observed, positioned at 498, 510, and 526 nm for BLa, BILa, and BDILa, respectively. These absorption peaks correspond to the characteristic strong S_0_→S_1_ (π→π *) transition of the boradiazaindacene chromophore [37,38]. In addition, a weaker and broad absorption band centered at approximately 378 nm was observed, which can be attributed to the out-of-plane vibrations of the aromatic skeleton S_0_→S_2_ (π→π*) transition [39,40]. While the absorption maxima of the BODIPY-based PS agents were in the range of 498–526 nm, they were deemed acceptable for the intended biological experiments. Furthermore, the emission spectra of the synthesized PS agents closely resembled their absorption spectra, demonstrating that the absorbing and emitting species possess similar corresponding structures. Moreover, the introduction of halogen atoms onto the BODIPY core, as in BILa and BDILa, induced noticeable red-shifts in the absorption (12–28 nm) and emission (14–32 nm) maxima, when compared to that of uniodinated BODIPY. This is owing to the heavy atom effect resulting from the incorporated halogen [15]. In addition, the emission peak of BODIPY dye BLa was centered at approximately 510 nm and its fluorescence quantum yield was calculated as being approximately 0.64. Iodination resulted in a red-shift of the emission peaks of BILa and BDILa to 524 nm and 542 nm, respectively, and a significant decrease in their respective fluorescence quantum yields was observed, compared to uniodinated BLa. The fluorescence quantum yields were determined as 0.06 and 0.02 for BILa and BDILa, respectively. Such a reduction in fluorescence quantum yields, as strongly influenced by the heavy atom effect, suggests improved photosensitizing capabilities in the iodinated BODIPY dyes BILa and BDILa in comparison to that of non-iodinated BLa.

**Figure 2:**
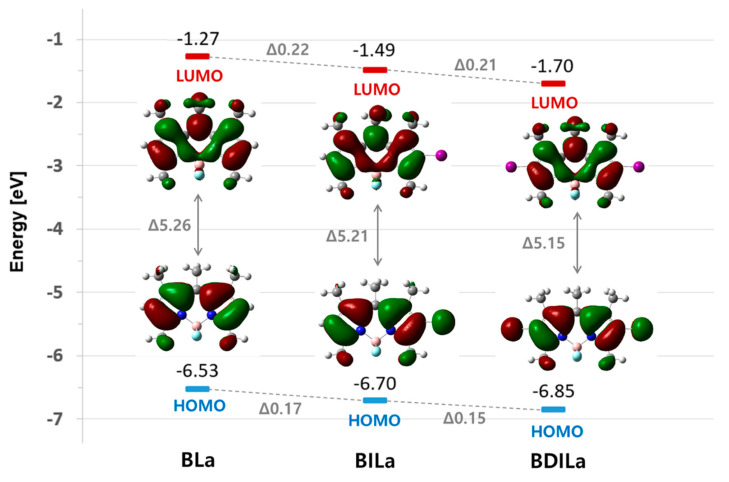
Highest occupied molecular orbital-lowest unoccupied molecular orbital (HOMO-LUMO) transition energies and wave function of BLa, BILa, and BDILa calculated using the density functional theory (DFT) method with the CAM-B3LYP functional and 6-31G(d,p) basis set (LanL2DZ basis for I atoms).

**Figure 3:**
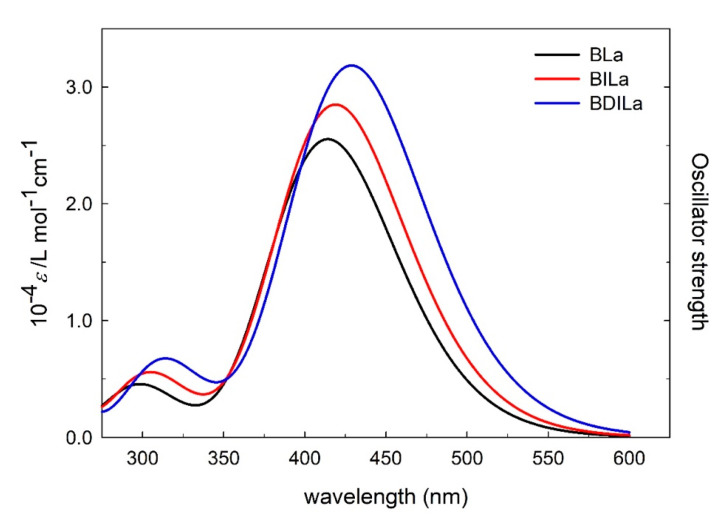
The simulated UV-Vis spectrum of BLa, BILa, and BDILa in water solution.

To further elucidate the electronic effects of iodination on BODIPY properties, frontier molecular orbitals (MOs) of the dyes were theoretically analyzed based on optimized molecular structures of the BODIPY derivatives. As shown in Figure 2 and Table 2, the narrowing of the highest occupied molecular orbital-lowest unoccupied molecular orbital (HOMO-LUMO) gap primarily arose due to a destabilization of the HOMO, caused by the substituted iodine atoms, resulting in increased oscillator strength and a red-shift of the absorption band. Moreover, a simulation of the BLa, BILa, and BDILa in water solution was carried out. As indicated in Figure 3, the calculated absorption spectra in the water solvent for the three compounds are blue-shifted when compared with that of the experimental values. Such shifts were also found to some reported BODIPY systems [40].

### 2.3. Singlet Oxygen Generation of Water-Soluble BODIPY Derivatives

While a crucial factor for determining the success of a PDT agent is its ability to subsequently produce ^1^O_2_ after an energy transfer occurs from the photosensitizer triplet excited state (T_1_) to the molecular oxygen, the majority of BODIPY-based PSs suffer from low singlet oxygen quantum yields, as an intramolecular electronic transition from a singlet to a triplet excited state is spin-forbidden. For most compounds, absorbed light energy is largely contained in singlet excited states (S_n_), rather than undergoing singlet-to-triplet ISC, and released as fluorescence [41]. A spin-orbit perturbation is generally required for an effective transition between states of different spin multiplicities to occur [42]. It has been well-documented that the direct incorporation of heavy atoms such as halogens onto the molecule, or confinement of the molecule within a heavy-atom-rich environment significantly enhances spin–orbit perturbations, influences the rates of the spin-forbidden electronic transition from a singlet to a triplet state (ISC), and facilitates the generation of ^1^O_2_ [43,44]. Hence, iodine atoms were introduced at the 2- and 2,6-positions of the BODIPY core and compared their ^1^O_2_ production capabilities to that of the uniodinated control, which relies solely on its inherent spin-orbit coupling property in this process. Herein, indirect ^1^O_2_ detection was performed to evaluate singlet oxygen generation of the BODIPY-based PS agents via photodegradation of the fluorescent dye, 1,3-diphenylisobenzofuran (DPBF). The mechanism of ^1^O_2_ detection by DPBF relies on its reaction with ^1^O_2_ to generate an endoperoxide through a [4 + 2] cycloaddition reaction. The endoperoxide subsequently decomposes to 1,2-dibenzoylbenzene, resulting in the total loss of the extended π-electron system and its distinctive spectroscopic characteristics [45]. The photooxidation rates of the quencher were tracked spectroscopically by monitoring the change in the absorption bands of DPBF at 424 nm [46].

**Figure 4:**
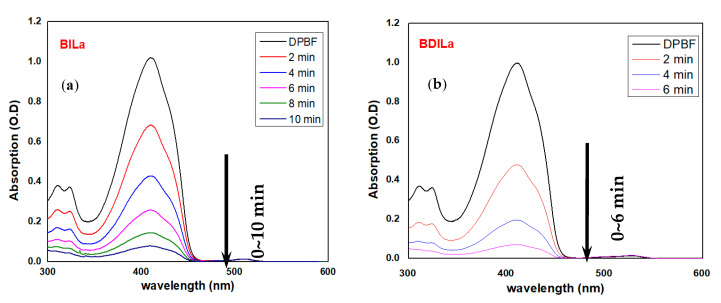
Time-dependent absorption spectra of 1,3-diphenylisobenzofuran (DPBF) in EtOH with (**a**) BILa and (**b**) BDILa after LED light excitation at 500 nm.

Trapping experiments were performed by the treatment of DPBF (50 μM) with BODIPY-based PS agents (0–2.0 μM) in an air-saturated EtOH medium and subsequent exposure of the resulting solutions to an LED (λ_max_ = 500 nm) light source, corresponding to an irradiance of 9 mW/cm^2^. As shown in Figure 4, extensive DPBF bleaching was observed after incubation with the mono- and diiodinated BODIPY dyes, BILa and BDILa, respectively, and LED irradiation. DPBF bleaching was manifested in the disappearance of its distinct absorbance band in the region of 424 nm. As shown in Figure 4a, the absorbance band for the monoiodinated dye BILa was nearly completely absent after 10 min of incubation, while its diiodinated counterpart, BDILa, exhibited a more abrupt disappearance of the absorbance band (~6 min, Figure 4b). In contrast, no significant photooxidation of DPBF was observed for the BLa control (Figure S1), as the distinct DPBF absorbance peak at around 424 nm was evident even after 70 min of continuous exposure to the light source.

The ^1^O_2_ quantum yields (Φ_Δ_) of the synthesized BODIPY derivatives were additionally determined using hematoporphyrin (HP) as a reference standard, the Φ_Δ_ of which is known (0.53 in EtOH). The decay curves of the absorption density and the linearly fitted degradation rates for DPBF in the presence of the test samples and HP are presented in Figure S2a,b, respectively. The Φ_Δ_ of the BODIPY dyes BILa and BDILa were calculated as 0.27 and 0.47, respectively. On the other hand, BLa could not generate ^1^O_2_ under the same experimental conditions (Φ_Δ_ = 0.01). These results collectively indicate that both iodinated-BODIPY dyes BILa and BDILa achieved elevated ^1^O_2_ generation under LED illumination. In the case of BDILa, the additional heavy iodine atom induced further spin–orbit perturbations, resulting in its superior capability to generate singlet oxygen, as evidenced by the faster diminishing rate of DPBF absorbance bands and the higher singlet oxygen quantum yield, compared to that of the monoiodinated dye BILa. Nevertheless, both PS agents exhibited the heavy atom effect and facilitated singlet oxygen generation, demonstrating their potential as efficient photosensitizers for PDT.

### 2.4. Assessment of Cytotoxicity of the Water-Soluble BODIPY Derivatives

Another critical factor to consider when evaluating the potential of a photosensitizer for clinical applications, particularly as a PDT agent for cancer treatment, is their ability to exhibit minimal cytotoxicity in the dark, but outstanding toxicity under light irradiation. Herein, the cytotoxicities of the synthesized water-soluble BODIPY-based PS agents were assessed against three distinct cancer cell lines (Huh7, HeLa, and MCF-7). Cell viability assessments were performed using [3-(4,5-dimethylthiazol-2-yl)-5-(3-carboxymethoxyphenyl)-2-(4-sulfophenyl)-2H-tetrazolium] (MTS), a substrate of mitochondrial succinate dehydrogenase (SDH, EC 1.3.5.1), which is reduced to formazan by mitochondrial activity of metabolically active cells. The quantity of formazan generated by dehydrogenase enzymes is directly proportional to the number of living cells in culture and can be measured spectroscopically at 490 nm [47]. The three cancer cell lines were incubated with various concentrations of the synthesized BODIPY dyes and the resulting cell viabilities were measured in the absence and presence of 530 nm LED light illumination. Then, the absorbance values of the wells containing solutions of MTS (background) were subtracted from those of the wells containing the treated and control cells. As shown in Figure S3, the toxicities in the dark of all the synthesized BODIPY-based photosensitizers were found to be negligible against Huh7, and the cells retained at least 96% viability even at a maximum dye concentration of 2.0 μM. The same low cytotoxicities in the dark were observed against the other two cancer cell lines, HeLa and MCF-7 (Figures S4 and S5, respectively, in the supporting information), wherein all three BODIPY dyes exhibited 96% cell viability or above, even after incubation at a maximum concentration (2.0 μM). The low toxicities exhibited by the synthesized PS agents in the absence of light irradiation are a possible indication of their favorable biocompatibility, attributable to the biological prevalence of the attached lactose moiety.

### 2.5. Cellular Uptake by Flow Cytometry

**Figure 5:**
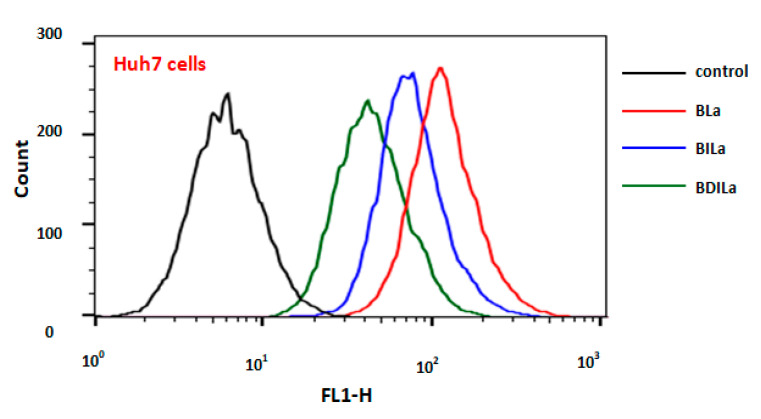
The fluorescence-activated cell sorting (FACS) analysis of the BODIPY dyes BLa, BILa, and BDILa in Huh7 cells.

The photosensitizer efficacy as a PDT agent is well-associated with its effective cellular uptake and subcellular localization [48,49]. Therefore, cellular uptake of the synthesized BODIPY derivatives was quantitatively investigated in Huh7, HeLa, and MCF-7 cells by fluorescence intensity measurements, employing flow cytometry. The cells were incubated with 2.0 μM of the BODIPY dyes at 37 °C. After 2 h of incubation, the treated cells were collected and subjected to fluorescence-activated cell sorting (FACS) analysis. As a control, untreated cell lines were likewise subjected to FACS analysis under the same experimental conditions. The corrected median fluorescence intensities (MFI) of the synthesized dyes against treated cancer cell lines were summarized in Table 3. As depicted in Figure 5, Figures S6 and S7, the corresponding controls (without photosensitizers) in all cell types displayed low fluorescence intensities that corresponded to their inherent mitochondrial autofluorescence, suggesting that the fluorescence intensities exhibited by the treated cancer cells were attributable to intracellularly accumulated PS agents. The relative cellular internalization of the PS agents across all cell types was BDILa < BILa < BLa.

The results for the iodinated-BODIPY dyes, BILa and BDILa compared with the corresponding parent dye BLa, clearly demonstrated that the incorporation of iodine atoms onto the BODIPY core decreased their capability for internalization by the treated cancer cells. For instance, Huh7 cells treated with BLa exhibited the highest fluorescence intensity (Figure 5), suggesting enhanced cellular uptake of the incorporated dye. The mean fluorescence for Huh7 cells treated with BLa was approximately 18-fold higher than that of the untreated cells, whereas BILa- and BDILa-treated Huh7 cells exhibited approximate increases of 12- and 7-fold, respectively. This trend was similarly observed for the other two cancer cell lines (Figures S6 and S7). Both HeLa and MCF-7 cells incubated with BDILa, BILa, and BLa demonstrated an increase in their mean fluorescence of approximately ~5-, ~9 to 11, and ~16-fold, respectively, relative to that of the untreated cells. The overall cellular uptake profiles of the PS agents agree with the rationale that molecular synthetic modifications can lead to significant variations in partition coefficient values. It is important to note that the overall lipophilicity/hydrophilicity of the photosensitizer plays a vital role in photosensitizer-cell surface interactions [50]. Hence, a favorable balance between the hydrophilicity and lipophilicity of the PS is imperative to achieve adequate biodistribution and cellular uptake. Excessive PS lipophilicity would impede its transport through the blood vessels, while high hydrophilicity would hamper its penetration into cell membranes [35]. While additional iodine atoms facilitate the efficient generation of singlet oxygen, they however decrease the overall hydrophilicity of the molecules, resulting in reduced uptake of the PS by the treated cells. Particularly, the diiodinated derivative BDILa exhibited relatively low cellular uptake, as evidenced by its lower fluorescence intensity, when compared to that of BILa and BLa. Despite the slight decrease in cellular uptake, both of the iodinated-BODIPY PS agents induced excellent phototoxicity towards the tested cancer cell lines, thus implying that the incurred cellular uptake is sufficient for photodynamic therapy.

It is also noteworthy that several proteins, receptors, and transporter molecules were found to be overly expressed on the tumor cell surfaces and membranes. Such overly expressed receptors and transporters are unique and specific for a given cell line. For instance, hepato liver carcinoma Huh7 cells are known to display overexpressed C-lectin type and asialoglycoproteins (ASGPR) receptors [51,52], while human breast adenocarcinoma MCF-7 cells possess mannose-receptor-rich tumor cell surfaces [18]. These overly expressed receptors can interact with the targeting molecules via carbohydrate-mediated cell recognition processes [24,25]. Hence, we compared the cellular uptake profiles among all the cancer cell lines tested, to assess the tumor-targeting capabilities of the PS agents. The lactose moiety of the synthesized BODIPY derivatives serves as the targeting ligand that can selectively interact with a specific overexpressed receptor on the tumor cell surface. The results indicated that Huh7 cells exhibited the highest transfection of the lactose-modified photosensitizers, compared to that of the other two cancer cell lines. As mentioned, the surfaces and membranes of Huh7 cells contained overexpressed C-lectin type and ASGPR receptors, which have been well-documented to selectively bind to and interact with galactose and galactose-functionalized macromolecules [53,54]. This may explain the higher transfection of the photosensitizers in Huh7 cells, attributable to enhanced carbohydrate-protein interactions, which demonstrates the favorable tumor-targeting capacity of the water-soluble lactose-modified BODIPY-based photosensitizers.

### 2.6. Photodynamic Anticancer Activity of the Water-Soluble BODIPY Derivatives

**Figure 6:**
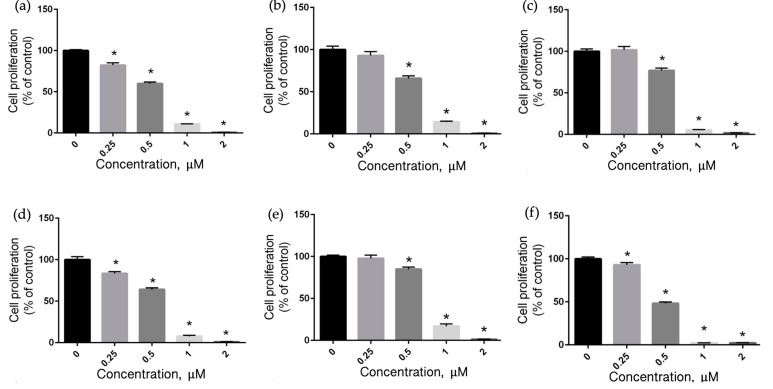
Dose-dependent cytotoxicities of synthesized BODIPYs. Cell survival rates of (**a**) HeLa, (**b**) MCF-7, and (**c**) Huh7 cells after treatment with BILa; (**d**) HeLa, (**e**) MCF-7, and (**f**) Huh7 cells after treatment with BDILa under LED light irradiation at 530 nm. * *p* < 0.05 compared to control (0 μM).

The photodynamic therapeutic potential of synthesized PS agents is contingent on their ability to generate ROS, particularly ^1^O_2,_ after light irradiation [55,56]. Hence, we next assessed the toxicity of the BODIPY dyes BLa, BILa, and BDILa following LED light irradiation (530 nm) for approximately 20 min. Both iodinated-BODIPY dyes, BILa and BDILa induced significant dose-dependent cytotoxic effects (*p* < 0.05) in all three cancer cell lines. In the case of HeLa cells (Figure 6a,d), both BILa and BDILa exhibited toxic effects even at the lowest tested concentration (0.25 μM), and the degree of cytotoxicity increased with their increasing concentration. HeLa cell viability plummeted to below 10% and approached 0%, after incubation with 1.0 μM and 2.0 μM of both dyes, respectively. The IC50 for BILa and BDILa against HeLa cells were estimated to be 0.53 μM and 0.55 μM, respectively. The same dose-dependent cytotoxicities were observed for MCF-7 cells (Figure 6b,e), in which the toxic effects of BILa and BDILa were evident at a concentration of 0.5 μM and the induced toxicities increased at higher concentrations. Such cytotoxicities against MCF-7 cells correspond to an IC50 of 0.56 μM and 0.61 μM for BODIPY dyes BILa and BDILa, respectively.

As illustrated in Figure 6c, the mono-iodinated BODIPY dye BILa produced relevant toxic effects (*p* < 0.05) to Huh7 cells at a concentration of 0.50 μM, while its diiodinated counterpart BDILa achieved significant toxic effects (*p* < 0.05) at a lower concentration of 0.25 μM (Figure 6f). Notably, the photo-killing efficacies of diiodinated-BODIPY BDILa at concentrations above 0.5 μM were over 1.5-fold higher than that of monoiodinated-BODIPY BILa for all tested tumor cells. Moreover, the calculated IC50 for BILa and BDILa against Huh7 cells were estimated to be 0.60 μM and 0.50 μM, respectively. This is not surprising, as the additional iodine atom in BDILa enables increased production of singlet oxygen compared to its monoiodinated counterpart, resulting in more potent photodynamic therapeutic properties. These PDT effects are consistent with fluorescence and singlet oxygen quantum yield results for the dyes, as shown in Table 1.

In contrast, the control BODIPY BLa was incapable of killing the tested tumor cells even in the presence of LED light (Figure S8), as evidenced by cell viabilities of 95% and above. No significant change was observed for both HeLa and Huh7 cells after treatment with BLa and subsequent irradiation with LED light. Although a significant decrease (*p* < 0.05) in MCF-7 cell viability was observed with BLa treatment, cell survival rates were still considered high (at least 80%) compared to that of the iodinated-BODIPY dyes BILa and BDILa. Overall, these findings demonstrated that the synthesized BODIPY dyes BILa and BDILa were biocompatible and non-toxic to cells in the absence of light, while incurring cytotoxicity upon LED light irradiation, due to the generation of reactive singlet oxygen species. The obtained photodynamic therapeutic levels of the synthesized water-soluble BODIPY derivatives are comparable to other reported PS agents for PDT application, including phtalocyanine- and porphyrin-based photosensitizers [57,58,59,60]. 

## 3. Materials and Methods

### 3.1. Materials

All reagents were obtained from commercial sources. D-lactose, acetic anhydride, *N*,*N*-dimethylformamide (DMF), trichloroacetonitrile, 1,8-diazabicyclo [5.4.0]undec-7-ene (DBU), boron trifluoride diethyl etherate (BF_3_·Et_2_O), propargyl alcohol, sodium metal, Dowex-50 resin (H+ form), 5-bromovaleryl chloride, 2,4-dimethyl pyrrole, triethylamine (TEA), *N*-iodosuccimide (NIS) were purchased from Sigma Aldrich (St. Louis, MO, USA). Sodium azide, sodium ascorbate, copper (II) sulfate pentahydrate, sodium hydroxide (NaOH), sulfuric acid (H_2_SO_4_), sodium hydrogen carbonate (NaHCO_3_), magnesium sulfate (MgSO_4_), and ammonium carbonate [(NH_4_)_2_CO_3_] were procured from Daejung Chemical (Gyeonggi-do, South Korea) and used without further purification. Ethyl acetate (EtOAc), dichloromethane (CH_2_Cl_2_), tetrahydrofuran (THF), methanol, and other solvents were of analytical grade and were dried under calcium hydride prior to use, except THF.

All compounds were characterized by ^1^H- and ^13^C-NMR spectroscopy on a Bruker AM 250 spectrometer (Billerica, MA, USA) and high-resolution electrospray ionization mass spectrometry (HR-ESI-MS) on a SYNAPT G2-Si high definition mass spectrometer (Waters, London, United Kingdom).

### 3.2. Synthesis of Lactose-Modified Water-Soluble BODIPY Derivatives

#### 3.2.1. Synthesis of the BODIPY Core

The BODIPY core was synthesized according to our previously reported procedure [61]. In a 250-mL dry, round-bottomed flask, 5-bromovaleryl chloride (1.14 mL, 8.52 mmol) and 2,4-dimethyl pyrrole (1.75 mL, 17.04 mmol) were dissolved in dry CH_2_Cl_2_ (100 mL) at room temperature and degassed with a stream of Ar gas for 2 min. The resulting mixture was refluxed for 2 h and the solvents were then removed *in vacuo*. The residual mixture was re-dissolved in a mixture of toluene and CH_2_Cl_2_ (10:1, *v*/*v*), then TEA (4.8 mL) and BF_3_·Et_2_O (4.2 mL) were added. After heating at 50 °C for 1.5 h, the solvents were evaporated and the crude product was purified by column chromatography to afford the BODIPY core as an orange solid (2.12 g, 65% yield).

^1^H-NMR (300 MHz, CDCl_3_, δ, ppm): 6.07 (s, 2H), 3.48–3.43 (t, 2H), 3.02–2.96 (t, 2H), 2.52 (s, 6H), 2.43 (s, 6H), 2.08–2.04 (m, 2H), 1.87–1.82 (m, 2H).

#### 3.2.2. Synthesis of Dye 1

The previously synthesized BODIPY core (120 mg, 0.313 mmol) was dissolved in DMF (10 mL) under Ar gas, followed by the addition of NaN_3_ (407 mg, 6.2 mmol). The mixture was then stirred at room temperature for 20 h. The resulting product was extracted with CH_2_Cl_2_ and washed successively with water and brine. The obtained organic phases were combined, dried over MgSO_4_, filtered and the solvent removed in vacuo. The crude product was purified by column chromatography to afford the BODIPY dye 1 as a bright-orange solid (102 mg, 94% yield).

^1^H-NMR (300MHz, CDCl_3_, δ, ppm): δ 6.07 (s, 2H), 3.39–3.35 (t, 2H), 3.01–2.95 (t, 2H), 2.52 (s, 6H), 2.42 (s, 6H), 1.78–1.75 (m, 4H).

#### 3.2.3. Synthesis of BODIPY Dyes 2a and 2b

BODIPY dye 2a and 2b were synthesized according to our previously described procedure [62]. A representative procedure is shown for BODIPY dye 2a. Briefly, BODIPY dye 1 (300 mg, 0.87 mmol) was dissolved in dried CH_2_Cl_2,_ followed by NIS (117 mg, 0.52 mmol) addition. The mixture was stirred at room temperature for 2 h and the solvent was then evaporated on a rotary evaporator. The crude product was purified by column chromatography to afford BODIPY dye 2a as an orange solid (319 mg, 78% yield).

^1^H-NMR (300MHz, CDCl_3_, δ, ppm): δ 6.13 (s, 1H), 3.47-3.43 (t, 2H), 3.01–2.97 (t, 2H), 2.60 (s, 3H), 2.53 (s, 3H), 2.46 (s, 3H), 2.43 (s, 3H), 2.08–2.03 (m, 2H), 1.84–1.79 (m, 2H).

The di-iodinated BODIPY Dye 2b was obtained in the same manner as the mono-iodinated BODIPY dye 2a, except that the NIS equivalents were doubled. The BODIPY dye 2b was obtained as a red solid (85% yield).

^1^H-NMR (300MHz, CDCl_3_, δ, ppm): δ 3.49–3.44 (t, 2H), 3.08–3.03 (t, 2H), 2.62 (s, 6H), 2.50 (s, 6H), 2.10–2.05 (m, 2H), 1.86–1.78 (m, 2H).

#### 3.2.4. General Procedure for the Preparation of Water-Soluble BODIPY Dyes BLa, BILa, and BDILa

A series of water-soluble BODIPY dyes BLa, BILa, and BDILa was prepared according to our previously reported procedure [63]. A representative process is described for BLa.

BLa: BODIPY dye 1 (55 mg, 0.16 mmol), lactose propargyl (67 mg, 0.175 mmol), NaAsc (158 mg, 0.797 mol), and CuSO_4_.5H_2_O (80 mg, 0.32 mmol) were dissolved in a mixture of THF/water (15/5 mL, *v*/*v*). Then, the resulting mixture was stirred for 24 h at room temperature, extracted with EtOAc and water three times, and dried over MgSO_4_. Following filtration and solvent removal on a rotary evaporator, the crude product was purified by recrystallization from MeOH/diethyl ether to afford a black-red solid (yield 60 mg, 52% yield).

^1^H-NMR (300 MHz, CDCl_3_, δ, ppm): δ 8.05 (s, 1H), 6.14 (s, 2H), 4.52–4.5 (t, 2H), 4.44–4.39 (m, 2H), 3.96–3.94 (m, 2H), 3.84–3.78(m, 4H), 3.76–3.73 (m, 2H), 3.70–3.67 (m, 4H), 3.61–3.58 (t, 2H), 3.05–3.03 (m, 2H), 2.45 (s, 6H), 2.39 (s, 6H), 1.64–1.61 (m, 4H)

^13^C-NMR (75 MHz, CD_3_OD, δ, ppm): 155.02, 146.86, 145.43, 142.22, 132.39, 125.46, 122.53, 104.87, 103.47, 80.57, 77.02, 76.1, 74.72, 74.38, 72.37, 70.07, 63.2, 62.26, 61.8, 55.03, 50.29, 32.71, 31.33, 30.7, 28.55, 23.64, 16.4, 14.26

HR-MS-ESI: *m*/*z* 748.3154, calcd mass for C_32_H_46_N_5_O_11_NaBF_2_ 748.3153

BILa: The BODIPY dye BILa was synthesized according to the above-detailed general procedure to afford the title product as an orange solid (47% yield).

^1^H-NMR (300 MHz, CDCl_3_, δ, ppm): δ 8.03 (s, 1H), 6.21 (s, 1H), 4.45–4.43 (t, 2H), 4.4–4.39 (m, 2H), 3.97–3.94 (m, 2H), 3.86–3.81 (m, 4H), 3.75–3.74 (m, 2H), 3.63–3.58 (m, 4H), 3.47–3.46 (t, 2H), 2.88–2.87 (t, 2H), 2.53 (s, 3H), 2.48 (s, 3H), 2.3 (s, 6H), 2.08–2.07 (m, 2H), 1.56–1.55 (m, 2H)

^13^C-NMR (75 MHz, CD_3_OD, δ, ppm): 164.92, 157.84, 153.67, 146.95, 145.64, 144.75, 141.76, 133.13, 131.89, 125.53, 124.11, 105.05, 103.47, 80.69, 77.0, 76.49, 76.25, 74.74, 72.47, 70.32, 63.1, 62.46, 61.92, 50.71, 31.65, 31.27, 29.52, 28.59, 18.73, 16.74, 14.8

HR-MS-ESI: *m*/*z* 874.2120, calcd mass for C_32_H_45_N_5_O_11_NaBF_2_I 874.2119

BDILa: The BODIPY dye BDILa was synthesized according to the above-detailed general procedure to afford the title product as a red solid (63% yield).

^1^H-NMR (300 MHz, CDCl_3_, δ, ppm): δ 8.05 (s, 1H), 4.43–4.41 (t, 2H), 4.38–4.36 (m, 2H), 3.91–3.8 (m, 5H), 3.78–3.76 (m, 2H), 3.7–3.66 (m, 5H), 3.47–3.44 (t, 2H), 3–2.88 (t, 2H), 2.55 (s, 2H), 2.39 (s, 6H), 2.14–2.1 (m, 2H), 1.61–1.59 (m, 2H)

^13^C-NMR (75 MHz, CD_3_OD, δ, ppm): 156.35, 147.21, 145.71, 144.12, 132.46, 126.1, 125.53, 105.13, 103.55, 101.87, 98.1, 93.72, 80.63, 77.02, 76.52, 76.28, 74.75, 73.16, 72.62, 71.31, 70.36, 62.47, 61.91, 56.58, 50.3, 32.12, 29.48, 27.8, 23.6, 19.24, 16.35, 14.42

HR-MS-ESI: *m*/*z* 1000.1086, calcd mass for C_32_H_44_N_5_O_11_NaBF_2_I_2_ 1000.1085

### 3.3. Measurement of Photophysical Properties

UV absorption spectra were recorded in a 1-cm path length quartz cuvette employing a double-beam UV-2800 Uv-vis spectrophotometer (Shimadzu, Kyoto, Japan) at room temperature. The steady-state emission spectra were acquired using a F-4500 steady-state fluorometer (Hitachi, Tokyo, Japan) with an Xenon arc lamp and a photomultiplier detection system. All spectra were measured at 300–700 nm, in triplicate, and corrected for background intensities by subtracting the spectra of pure solvent measured under identical conditions.

### 3.4. Fluorescence Quantum Yield Measurements

The relative fluorescence quantum yields of BODIPY BLa, BILa, and BDILa were obtained by comparing the area under the corrected emission spectrum of the samples with that of a standard solution with a known fluorescence quantum yield. Herein, Rhodamine 6G was used as the reference standard, which possesses a known quantum yield of 0.94 in methanol [64]. The Φ_f_ was calculated according to Equation (1):

(1) $$\Phi_{S}=\Phi_{R}\;\left(\frac{Grad_{S}}{Grad_{R}}\right){\left(\frac{n_{S}}{n_{R}}\right)}^{2}$$

where the subscripts *S* and *R* represent the tested sample and reference, respectively. In addition, Grad and *n* denote the gradient of the filled slope and the refractive index of the test solvent used, respectively. The solutions were optically diluted to avoid inner filter effects [65].

### 3.5. Singlet Oxygen Quantum Yield Measurements

The sample quantum yields of singlet oxygen (Φ_Δ_) were studied using 1,3-diphenylisobenzofuran (DBPF) as a chemical quencher [46]. Briefly, a mixture of each BODIPY dye (absorption ~0.06 at 524 nm in EtOH) and DPBF (absorption ~1.0 at 424 nm in EtOH) was irradiated with a green LED lamp (λ_max_ = 500 nm). The photooxidation of DPBF was then monitored between 0 and 70 min, depending on the efficiency of the BODIPY dye. The singlet oxygen quantum yield was calculated using hematoporphyrin (HP) as the reference, with a yield of 0.53 in ethanol, according to the following equation:

(2) $$\Phi_{\Delta }^{S}=\frac{k_{S}}{k_{R}}\times \Phi_{\Delta }^{R}$$

where subscripts *S* and *R* represent the sample and reference, respectively, while *k* represents the slope of the photodegradation rate.

### 3.6. Cells and Cell Cultures

The three cancer cell lines, namely human cervix adenocarcinoma (HeLa), human breast adenocarcinoma (MCF-7), and hepato liver carcinoma (Huh7), were obtained from Korean Cell Line Bank and maintained according to the provider’s instructions. Briefly, the cell lines were cultured under standard culture conditions (5% CO_2_ and 95% air at 37 °C) in an RPMI 1640 medium (Gibco, Carlsbad, CA, USA) supplemented with 10% heat-inactivated fetal bovine serum (FBS) and antibiotic (100 U/mL penicillin and 100 mg/mL streptomycin) (WELGENE Inc., Gyeongsangbuk-do, Korea).

### 3.7. Cell Proliferation Assay

HeLa, MCF-7 and Huh7 cells were seeded in 96-well plates (3 × 103 cells/well). The cells were maintained for 24 h and treated with a range of test compound concentrations (0, 0.25, 0.5, 1.0 and 2.0 µM) for 24 h [66,67]. A cell proliferation assay was measured via CellTiter 96^®^ AQueous one solution cell proliferation assay (Promega, Madison, WI, USA) according to the manufacturer’s instructions. The absorbance was determined at 490 nm using an enzyme-linked immunosorbent assay (ELISA) plate reader (Thermo Fisher Scientific, Inc., Waltham, MA, USA).

### 3.8. Photodynamic Anticancer Activity Assessment

Pre-cultured cancer cells were plated at 3 × 103 cells/well in a 96-well plate and incubated at 37 °C in 5% CO_2_ for 24 h. The media was then replaced with fresh media and the cells were treated with various concentrations of BLa, BILa, and BDILa (0, 0.25, 0.5, 1.0 and 2.0 μM) at 37 °C in 5% CO_2_ for 2 h under dark conditions. Then, the media in all plates were changed to a phenol-red free RPMI 1640 media and the cells were irradiated with a green light-emitting diode (LED), which had a wavelength of 530 nm (80%, 20 min), as previously described [66,67]. The irradiation power of the LED was approximately 9 mW. The cells were then incubated for further 24 h post-irradiation with the LED, and the viable cells were measured using a CellTiter 96^®^ AQueous One Solution Cell Proliferation Assay according to the manufacturer’s instruction.

### 3.9. Cellular Uptake by Flow Cytometry

To investigate the cellular uptake of the samples by HeLa, MCF-7 and Huh7 cells, flow cytometry was performed. Briefly, each cell line was seeded at 1 × 105 cells/well in a 6-well plate and incubated at 37 °C in 5% CO_2_ for 24 h. The incubated cells were then treated with the test samples (2.0 µM). After 2 h, the cells were collected and analyzed using an FC500 flow cytometer (Beckman coulter, CA, USA).

### 3.10. Theoretical Calculations

The molecular structures of the BODIPY derivatives were optimized using density functional theory (DFT), CAM-B3LYP function and 6-31G(d,p) basis set (LanL2DZ basis for I atoms) in a stepwise manner, and then the electronic states of the BODIPY derivatives were calculated using time-dependent DFT (TD-DFT) with the CAM-B3LYP function and 6-31G(d,p) basis set (LanL2DZ basis for I atoms), adding the water solvent environment, on a supercomputer to obtain information regarding excited states.

### 3.11. Statistical Analysis

All data were expressed as the mean ± standard deviation and compared by one-way analysis of variance (ANOVA), followed by the Tukey’s multiple comparison test, using a Prism GraphPad 6 software (San Diego, CA, USA). A *p* value of < 0.05 was considered statistically significant in all analyses.

## 4. Conclusions

In summary, we prepared a series of water-soluble BODIPY derivatives bearing a lactose moiety via a facile and efficient copper iodide (CuI) catalyzed azide-alkyne cycloaddition (CuAAC) click reaction. The photophysical and biological properties of the synthesized BODIPY derivatives were extensively investigated and the dyes were tested as photosensitizers in photodynamic therapy. The incorporation of a heavy iodine atom onto the BODIPY core facilitated the efficient generation of single oxygen species. Moreover, the synthesized BODIPY-based PS agents exhibited no toxicity to the three tested cancer cell lines (HeLa, Huh7, and MCF-7), in the absence of light irradiation. Relative to the dye BLa, the halogenated BODIPY derivatives BILa and BDILa exhibited superior cytotoxic effects against the tested cancer cell lines after LED light irradiation, demonstrating their capacities as potent photosensitizers for PDT applications. The synthesized dyes were also effectively internalized by the tested cell lines, particularly by Huh7 cells. Such effective cellular uptake is attributable to carbohydrate-mediated recognition processes and interactions between the targeting biomolecule lactose, and overexpressed specific receptors on the tumor cells. The study presented herein offers an important contribution in the field of cancer treatment, by providing a simple and reliant synthetic route for generating water-soluble BODIPY-based photosensitizers with excellent biocompatibilities, adequate tumor-targeting abilities, high cellular transfection capacities, and effective photodynamic therapeutic capabilities.

## Figures and Tables

**Table 1:** Photophysical and parameters of the dyes BLa, BILa and BDILa.

	BLa	BILa	BDILa
λ_ab_ (nm) ^a^	498	510	526
λ_em_ (nm) ^a^	510	524	542
Φ_F_^b^	0.64	0.06	0.02
Φ_Δ_^c^	0.01	0.27	0.47
ε (M^−1^ cm^−1^)	56,000	51,600	41,800

^a^ in aqueous solution; ^b^ in methanolic solution; ^c^ in ethanolic solution.

**Table 2:** Selected transition energies and wave function of BIa, BILa, and BDILa in the water solution.

	Excited State	Energy [eV]	*λ* [nm]	*f* ^a^
BLa ^b^	S1	2.995	414	0.63
BILa ^c^	S1	2.959	419	0.70
BDILa ^c^	S1	2.890	429	0.79

^a^ Oscillator strength; ^b^ TDDFT method with the CAM-B3LYP function and 6-31G(d,p) basis set; ^c^ TDDFT method with the CAM-B3LYP function and 6-31G(d,p) basis set (LanL2DZ basis for I atoms) adding the water solvent environment.

**Table 3:** Corrected median fluorescence intensities (MFI) of the dyes BLa, BILa and BDILa against treated cancer cell lines.

	BLa (log) ^a^	BILa (log) ^a^	BDILa (log) ^a^
HeLa	7807 ± 460	5102 ± 123	2380 ± 100
Huh7	12083 ± 632	7589 ± 84	4138 ± 70
MCF-7	38952 ± 1730	5414 ± 447	2938 ± 219

^a^ Data are mean ± SD (*n* = 3).

## Supplementary Materials

The following are available online at https://www.mdpi.com/1420-3049/25/15/3340/s1, Figure S1: Time-dependent absorption spectra of the DPBF in EtOH solution with BLa after LED light excitation at 500 nm. Figure S2: (a) Normalized decay curves of the absorption density at λex = 424 nm for the DPBF in the presence of the BILa and BDILa against HP (normalized by the absorbance intensity at *t* = 0 min). (b) linearly fitted degradation rates for the DPBF in the presence of the test samples and HP. Figure S3: Cell survival rates of Huh7 cells after treatment with (a) BLa, (b) BILa, and (c) BDILa under dark conditions. Figure S4: Cell survival rates of HeLa cells after treatment with (a) BLa, (b) BILa, and (c) BDILa under dark conditions. Figure S5: Cell survival rates of MCF-7 cells after treatment with (a) BLa, (b) BILa, and (c) BDILa under dark conditions. Figure S6: The FACS analysis of the BODIPY dyes BLa, BILa, and BDILa in Hela cells. Figure S7: The FACS analysis of the BODIPY dyes BLa, BILa, and BDILa in MCF-7 cells. Figure S8: Cell survival rates of (a) HeLa, (b) MCF-7, and (c) Huh7 cells after treatment with BLa under LED light irradiation at 530 nm.
