# Supplementary material for: Synthesis and Photophysical Properties of Tumor-Targeted Water-Soluble BODIPY Photosensitizers for Photodynamic Therapy
Source: Molecules. 2020 Jul 23;25(15):3340. doi: 10.3390/molecules25153340 (PMC7435441; doi:10.3390/molecules25153340)
Supplement: Supplementary file 1 [file molecules-25-03340-s001.pdf]

molecules

Supplementary information

Synthesis and Photophysical Properties of Tumor-Targeted Water-Soluble BODIPY photosensitizers for Photodynamic Therapy

Duy Khuong Mai [1,2,†], Byungman Kang [3,†], Temmy Pegarro Vales [1,4,†], Isabel Wen Badon [1], Sung Cho [2,*], Joomin Lee [5,*], Eunae Kim [6,*] and Ho-Joong Kim [1,*]

[1] Department of Chemistry, Chosun University, Gwangju 61452, Korea; maikhuongduy@gmail.com (D.K.M.); valestemmy@gmail.com (T.P.V.); isabel.badon.isabel@gmail.com (I.W.B.)
[2] Department of Chemistry, Chonnam National University, Gwangju 61186, Korea
[3] Nuclear Chemistry Research Division, Korea Atomic Energy Research Institute, 989-111 Daedeok-daero, Yuseong-gu, Daejeon, 34057, Korea; alchem95@gmail.com
[4] Department of Natural Sciences, Caraga State University, Butuan City 8600, Philippines
[5] College of Food and Nutrition, Chosun University, Gwangju 61452, Korea
[6] College of Pharmacy, Chosun University, Gwangju 61452, Republic of Korea
* Correspondence: scho@chonnam.ac.kr (S.C.); joominlee@chosun.ac.kr (J.L.); eunaekim@chosun.ac.kr (E.K.); hjkim@chosun.ac.kr (H.-J.K.)
† These authors equally contributed to this work.

Academic Editors: M. Amparo F. Faustino, Carlos J. P. Monteiro and Catarina I. V. Ramos

Received: 30 June 2020; Accepted: 21 July 2020; Published: date

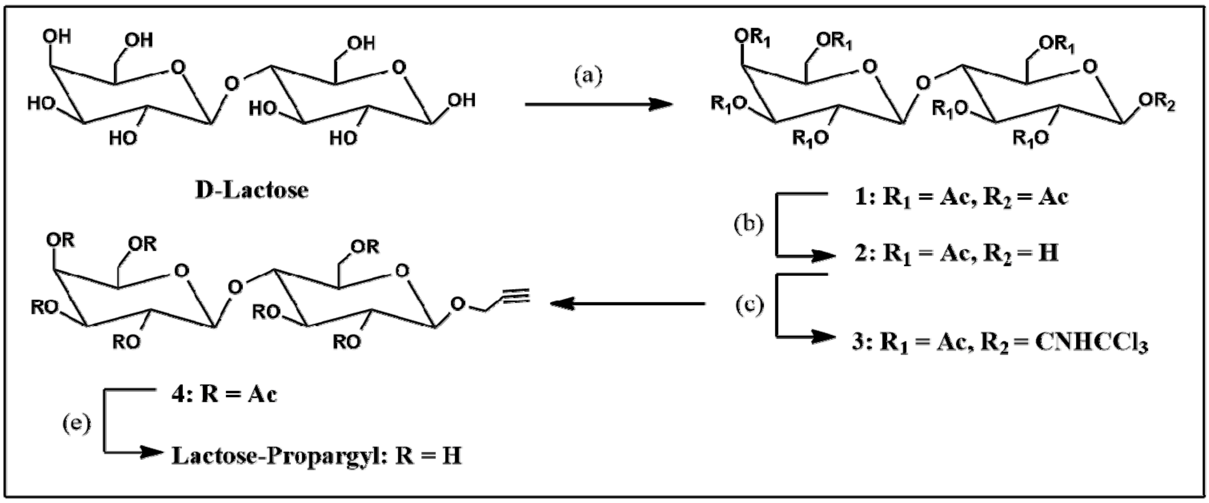

Scheme S1. The preparation of Lactose-Propargyl. *(a) Acetic anhydride, H_2SO_4, MC, rt. (b) $(NH_4)_2CO_3$, DMF, rt-24 h. (c) Trichloroacetonitrile, DBU, MC, 0 °C, 2 h. (d) Propargyl alcohol, $BF_3.Et_2O$, MC, 0 °C, 2 h. (e) NaOMe, MeOH, rt, 1h.*

D-Lactose Octaacetate (1) was carefully synthesized by following the reported method by Geng, et. al [1].
O-2,3,4,6-Tetra-O-acetyl-α,D-mannopyranosyl-(1->2)-3,4,6-tri-O-acetyl-α,D-mannopyranosyl-trichloroacetimidate (3). Imidate-contaning sugar was prepared according to the method of Matsuo, et.al with slight modification [2]. The compound **1** was reacted with $(NH_4)_2CO_3$ (19 g) in DMF (50 mL) at room temperature for 1 day. The mixture was then extracted with EtOAc (3 x 100 mL). The organic layers were collected and evaporated to afford the hemiacetal derivatives **2** without purification. In an ice-bath, the compound **2** and trichloroacetonitrile (10 eq) were dissolved in 30 mL of CH_2Cl_2 and subsequently added with the DBU (0.1 eq). The resulting mixture was stirred for 2 h and purified by column chromatography to afford compound **3**.
Propargyl derivative (4) was produced by the reported literature [3]. Briefly, $BF_3.Et_2O$ (0.24 mL, 1.85 mmol) was added to the mixture of the compound **3** (9.04 g, 18.5 mmol) and propargyl alcohol (1.1 mL, 19 mmol) in dry CH_2Cl_2 (100 mL) in an ice-bath under Argon for 2 h. The mixture was quenched by aq. $NaHCO_3$ and filtered by Celite. The organic layer was washed with NaOH (1M) and evaporated to yield the yellow oil. The sugar derivative **4** was afforded by recrystallization as a white solid.

Lactose-Propargyl
Lactose-Propargyl was synthesized according to the method was reported by Peet, et.al [3]. The propargyl **4** was dissolved in MeOH (30 mL) and a small piece of sodium metal was then added to the solution. After stirring at room temperature for 1 h, Dowex-50 resin (H+ form) was added until the mixture became

neutral. The mixture was then filtered, the solvent was evaporated to yield a solid. By careful recrystallization, the lactose-propargyl was afforded as colorless crystals.

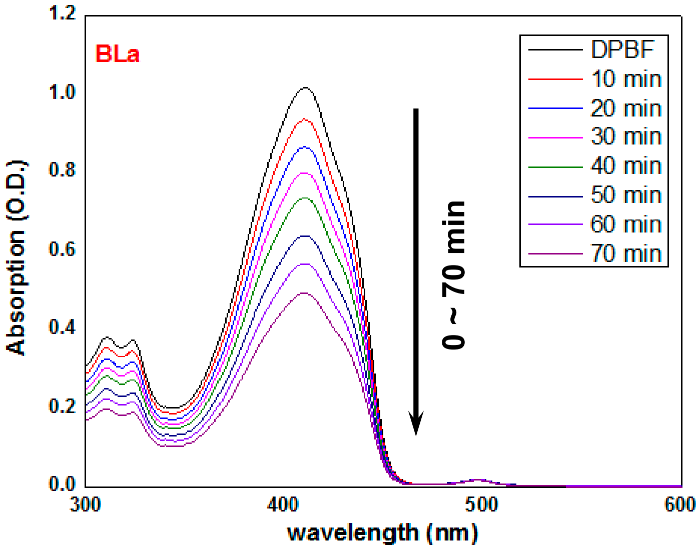

Figure S1. Time-dependent absorption spectra of the DPBF in EtOH solution with BLa after LED light excitation at 500 nm.

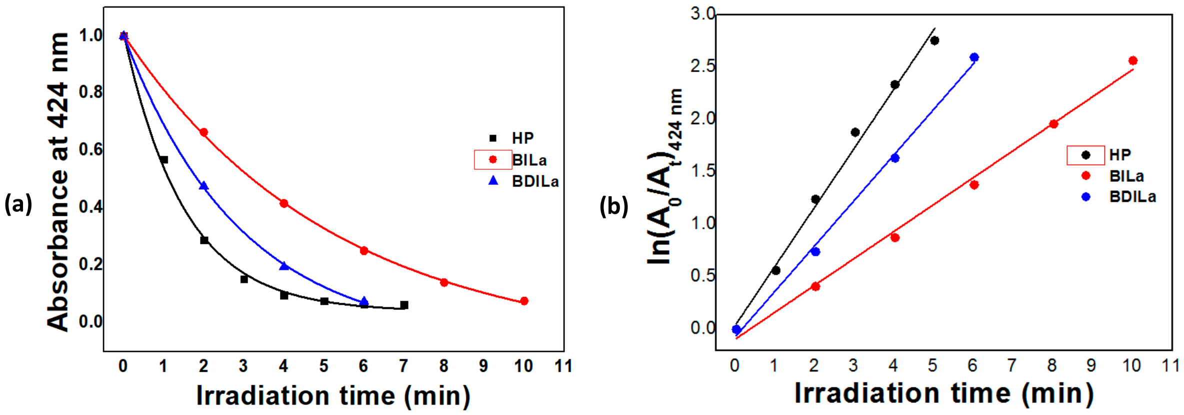

Figure S2. (a) Normalized decay curves of the absorption density at λ_{ex}=424 nm for the DPBF in the presence of the **BILa** and **BDILa** against HP (normalized by the absorbance intensity at t=0 min). (b) linearly fitted degradation rates for the DPBF in the presence of the test samples and HP.

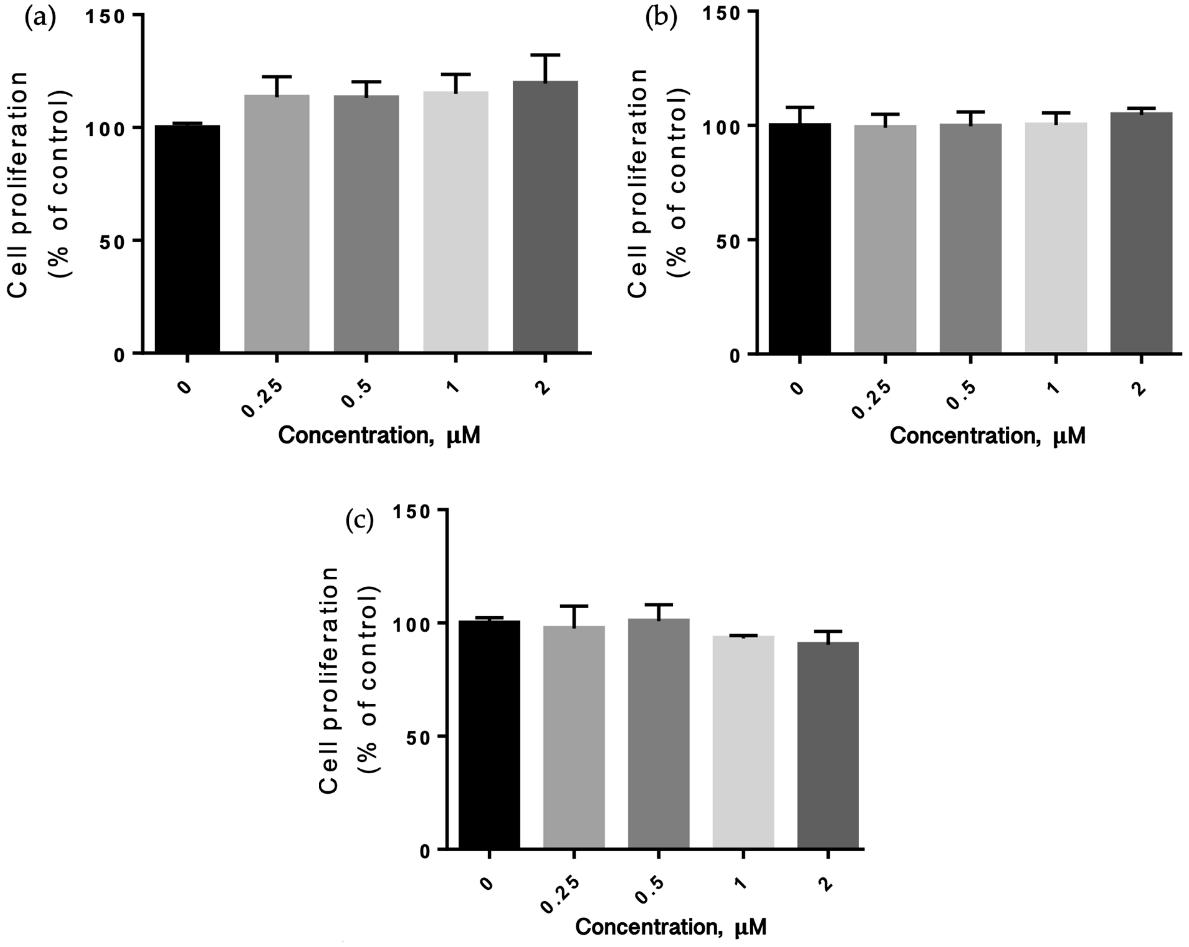

Figure S3. Cell survival rates of Huh7 cells after treatment with (a) BLa, (b) BILa, and (c) BDILa under dark conditions.

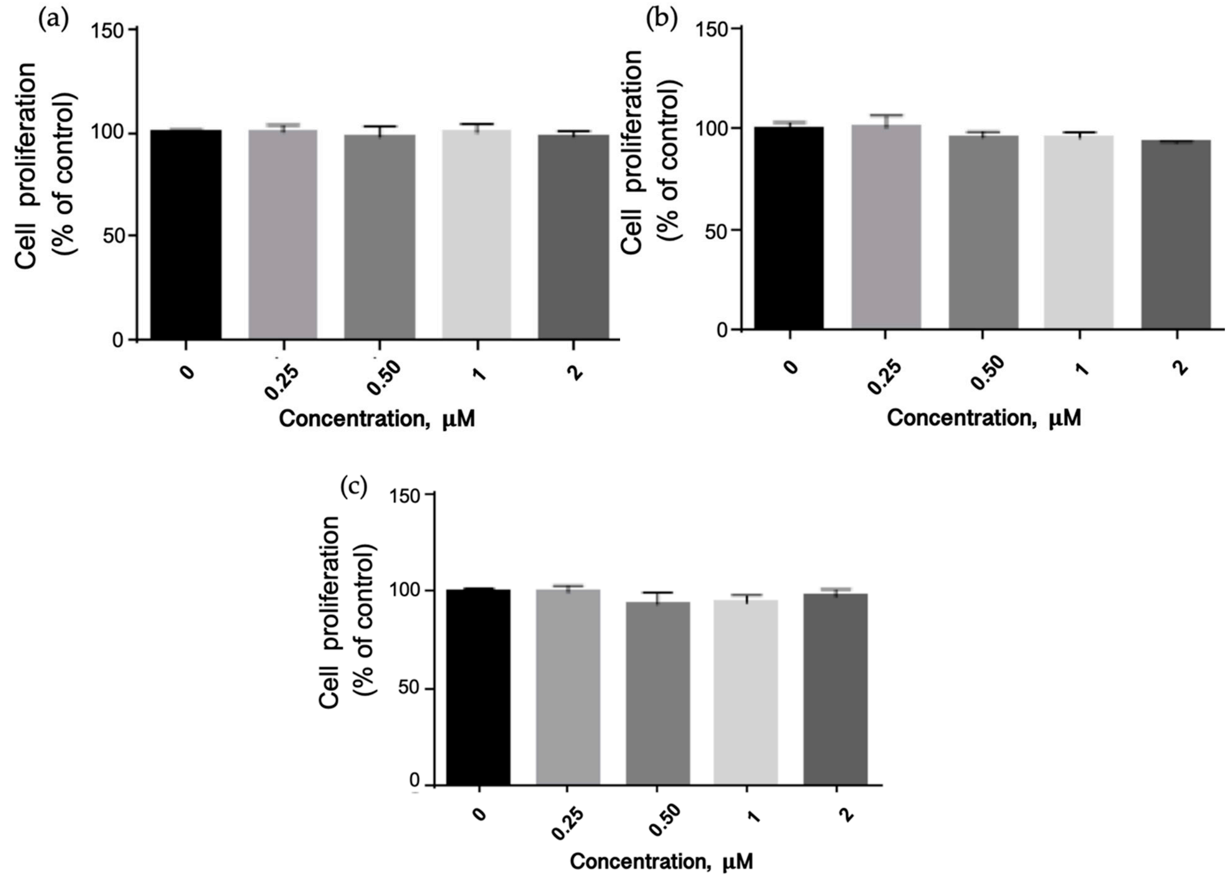

Figure S4. Cell survival rates of HeLa cells after treatment with (a) BLa, (b) BILa, and (c) BDILa under dark conditions.

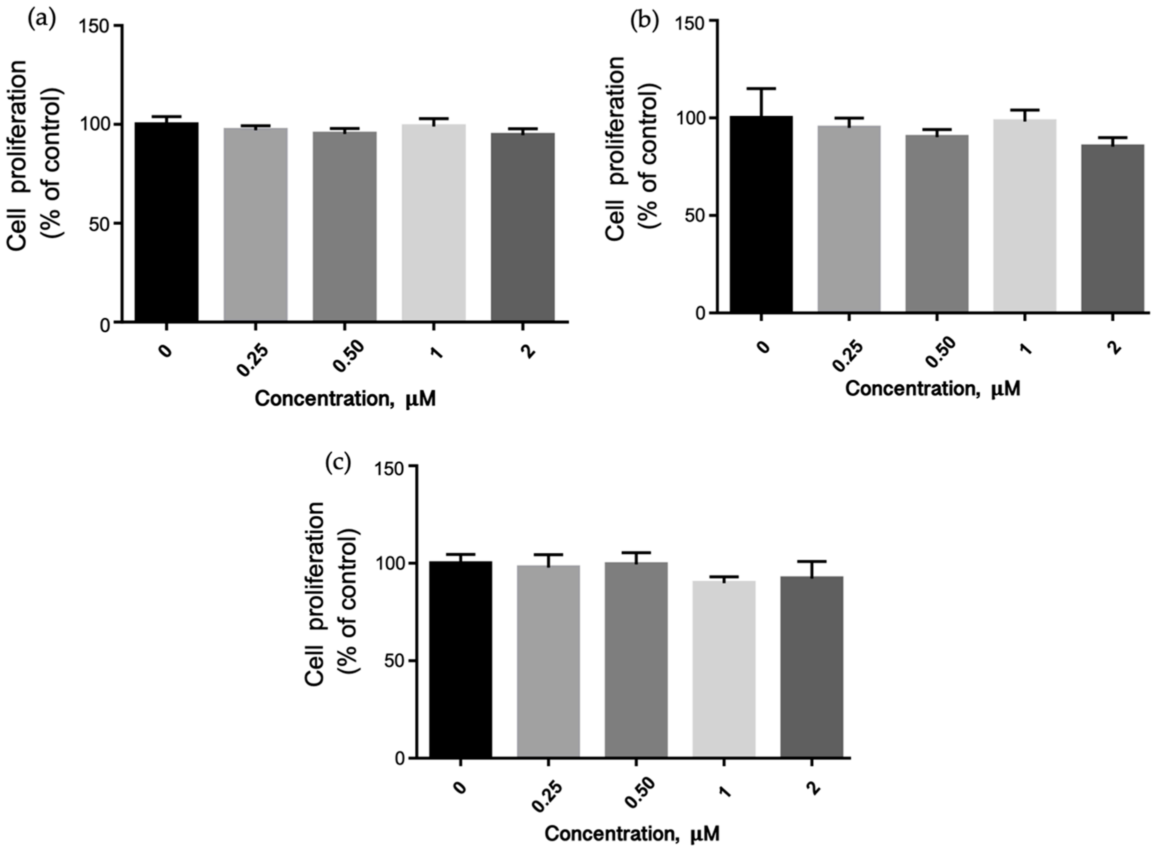

Figure S5. Cell survival rates of MCF-7 cells after treatment with (a) BLa, (b) BILa, and (c) BDILa under dark conditions.

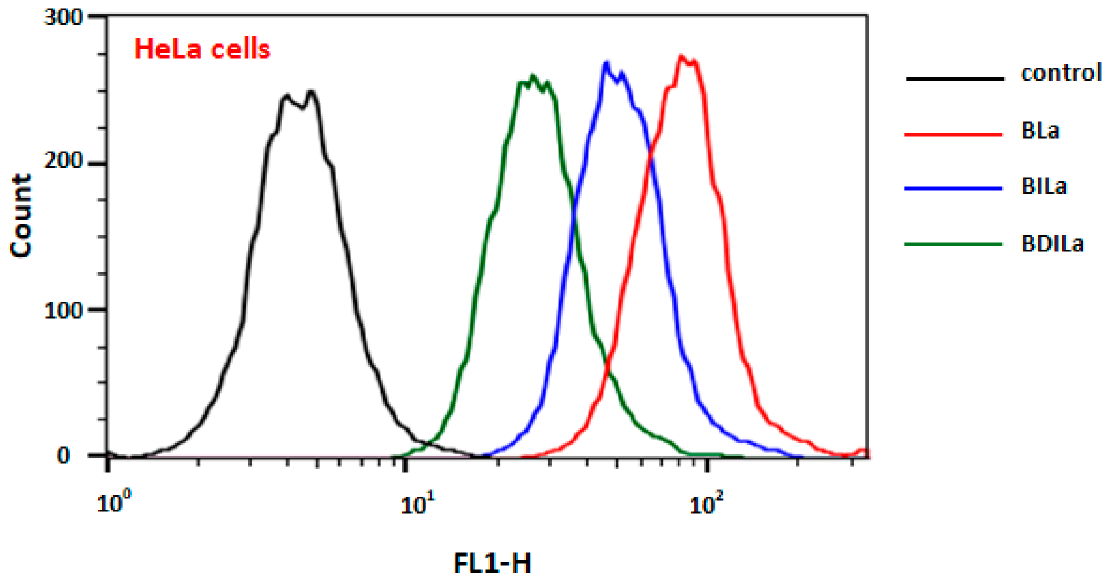

Figure S6. The FACS analysis of the BODIPY dyes **BLa, BILa,** and **BDILa** in Hela cells.

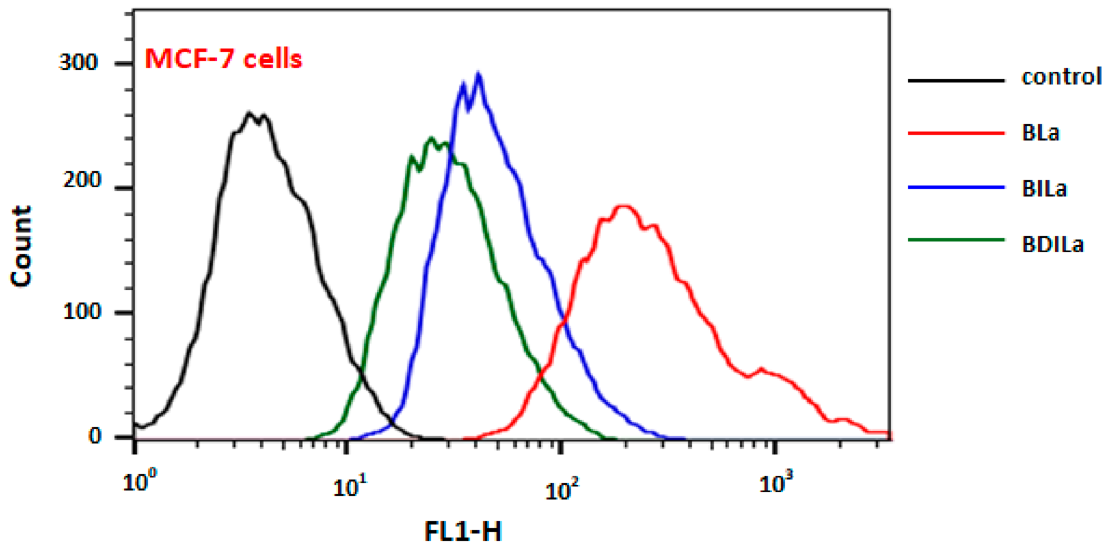

Figure S7. The FACS analysis of the BODIPY dyes **BLa, BILa,** and **BDILa** in MCF-7 cells.

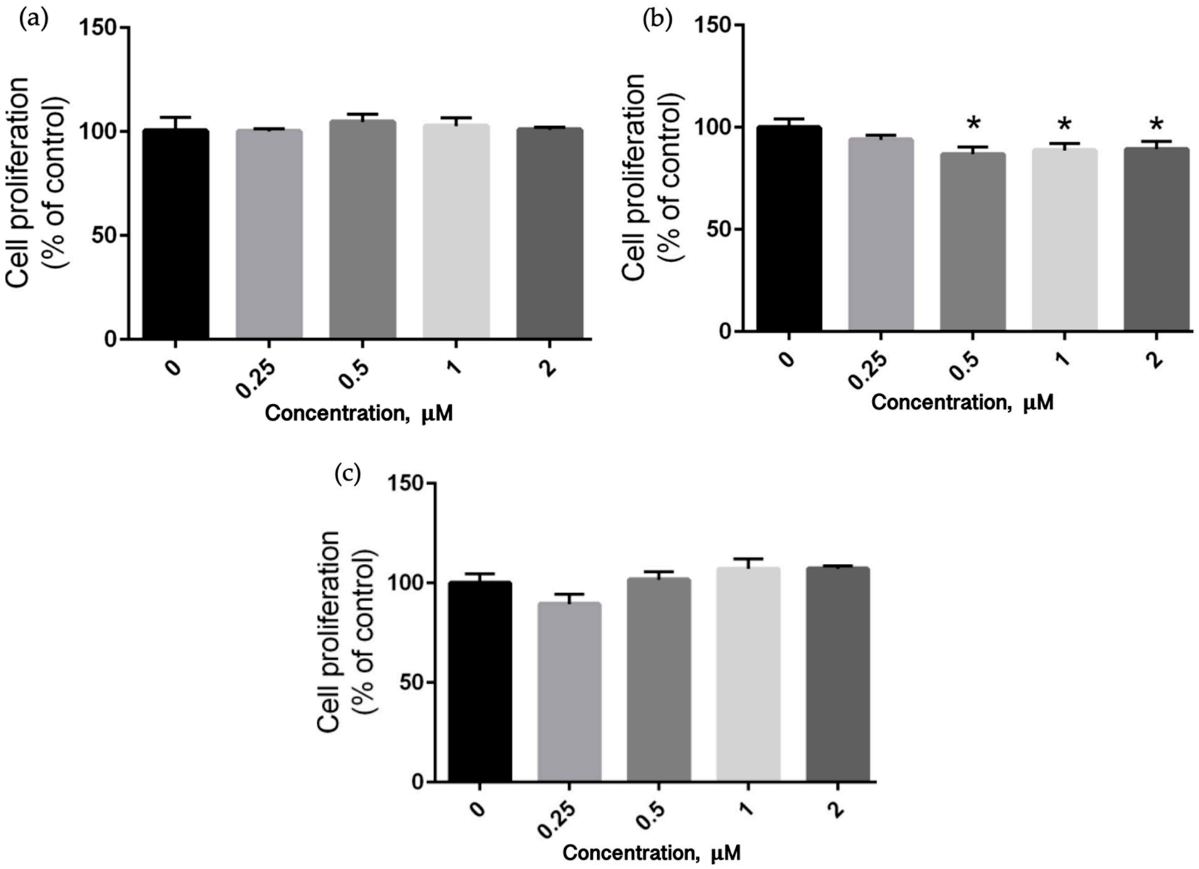

Figure S8. Cell survival rates of (a) HeLa, (b) MCF-7, and (c) Huh7 cells after treatment with BLa under LED light irradiation at 530 nm.

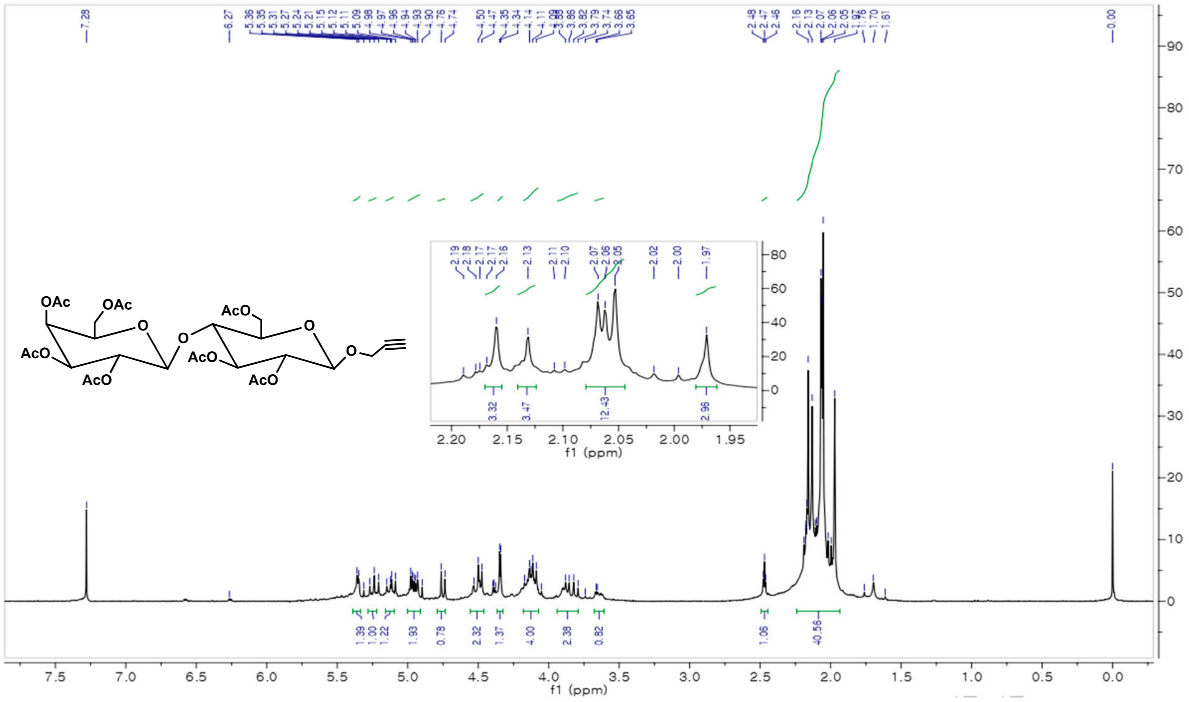

Figure S9. ^{1}H-NMR spectrum of propargyl derivative (4).

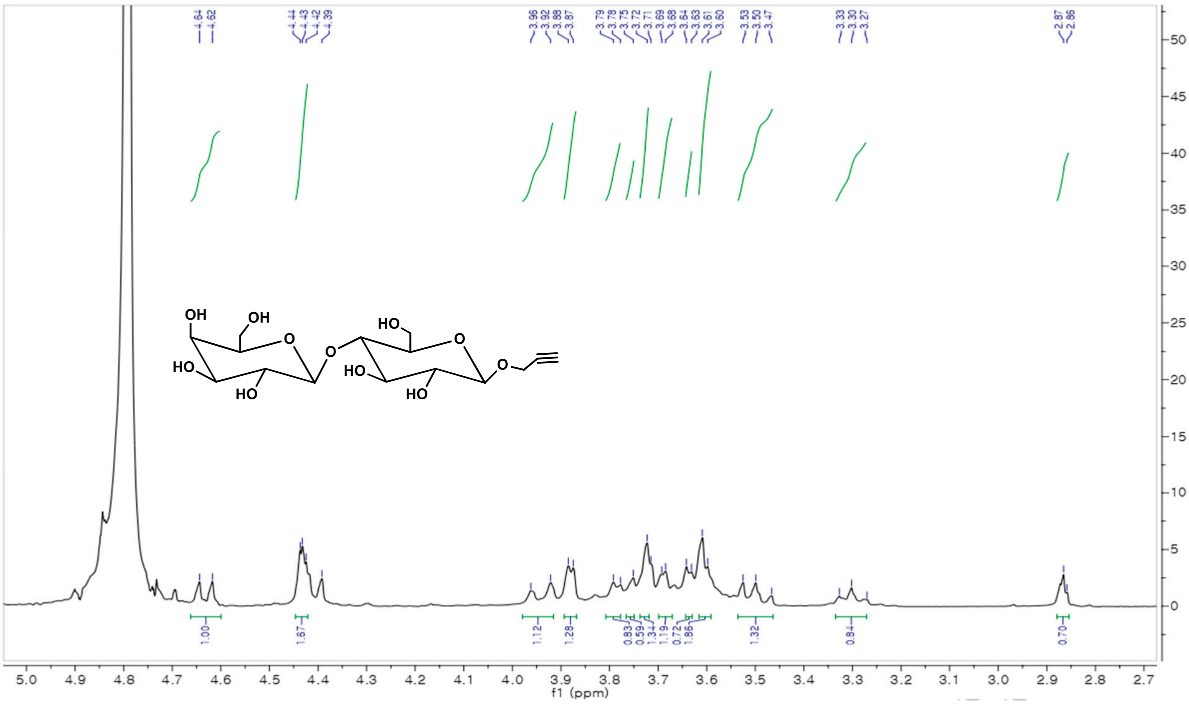

Figure S10. ^{1}H-NMR spectrum of lactose-propargyl.

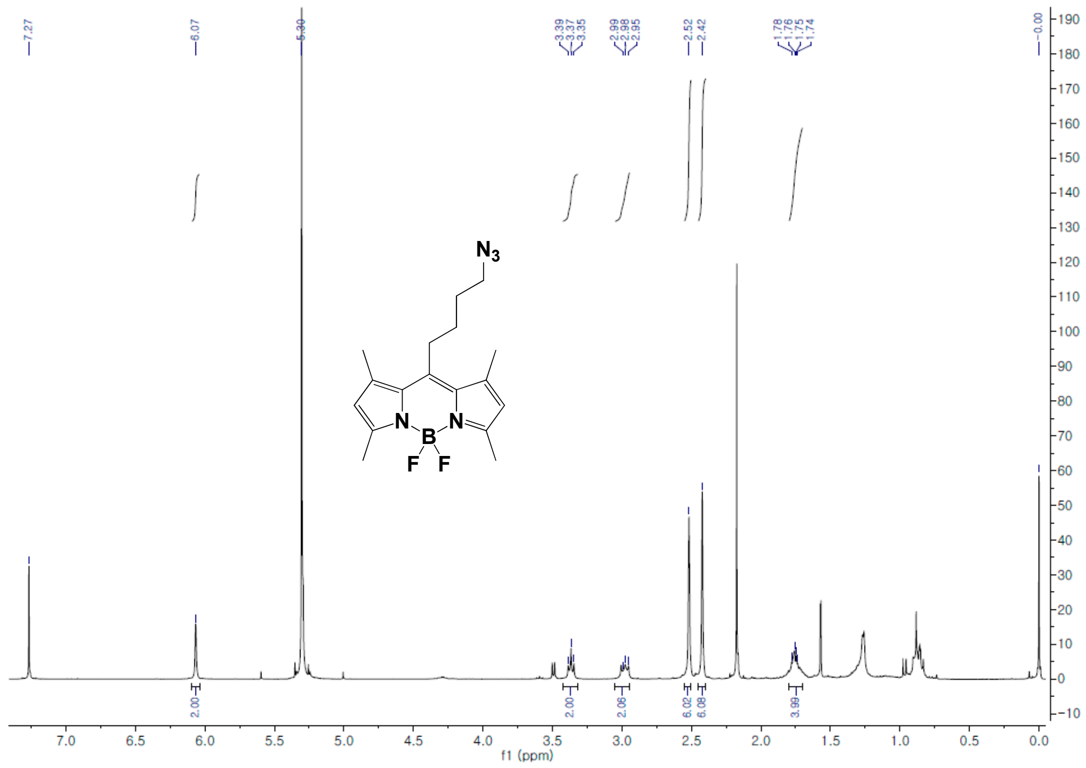

Figure S11. ^{1}H-NMR spectrum of BODIPY derivative 1.

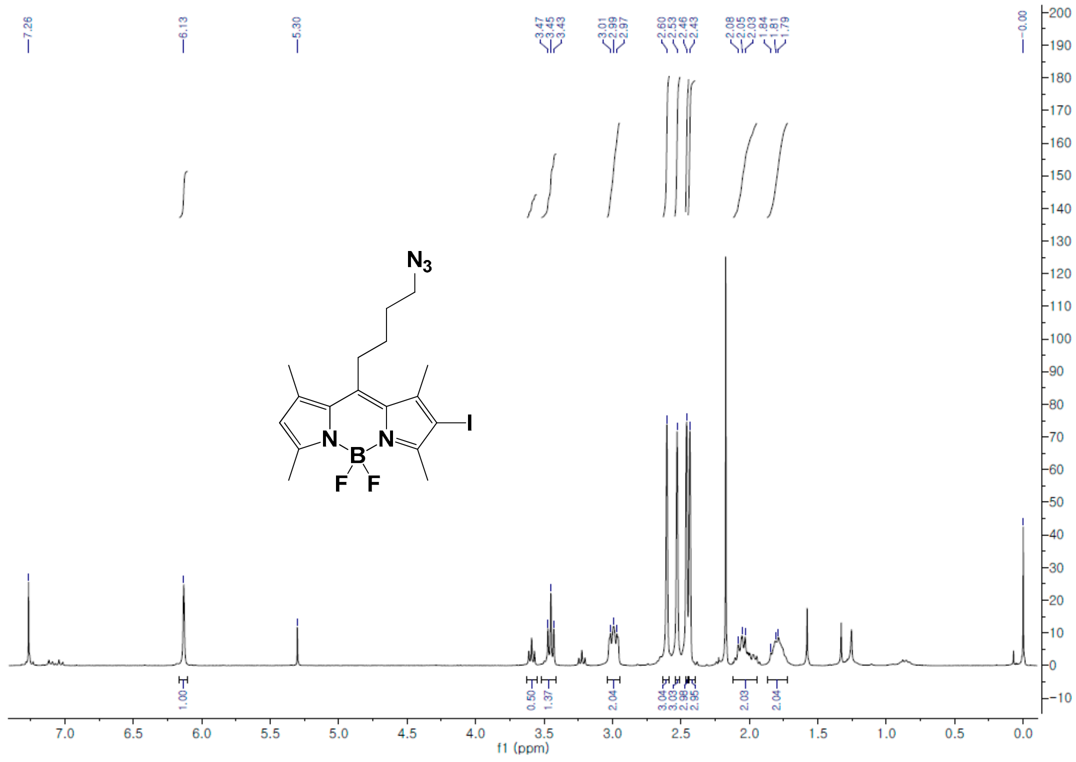

Figure S12. ^{1}H-NMR spectrum of BODIPY derivative 2a.

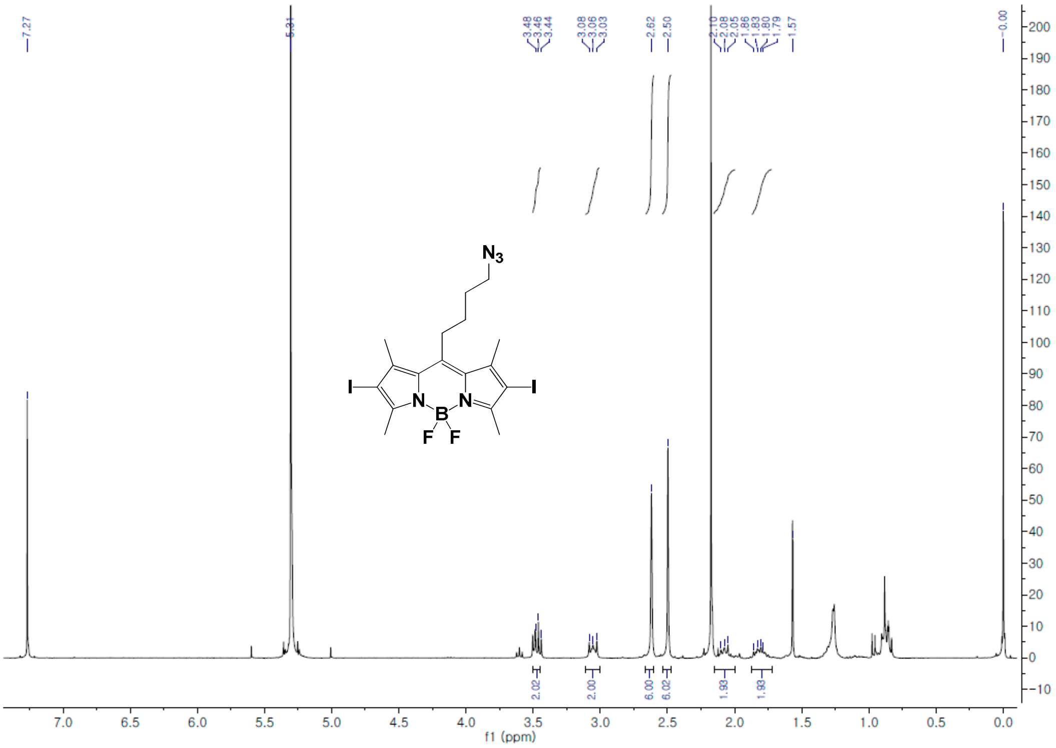

Figure S13. ^{1}H-NMR spectrum of BODIPY derivative 2b.

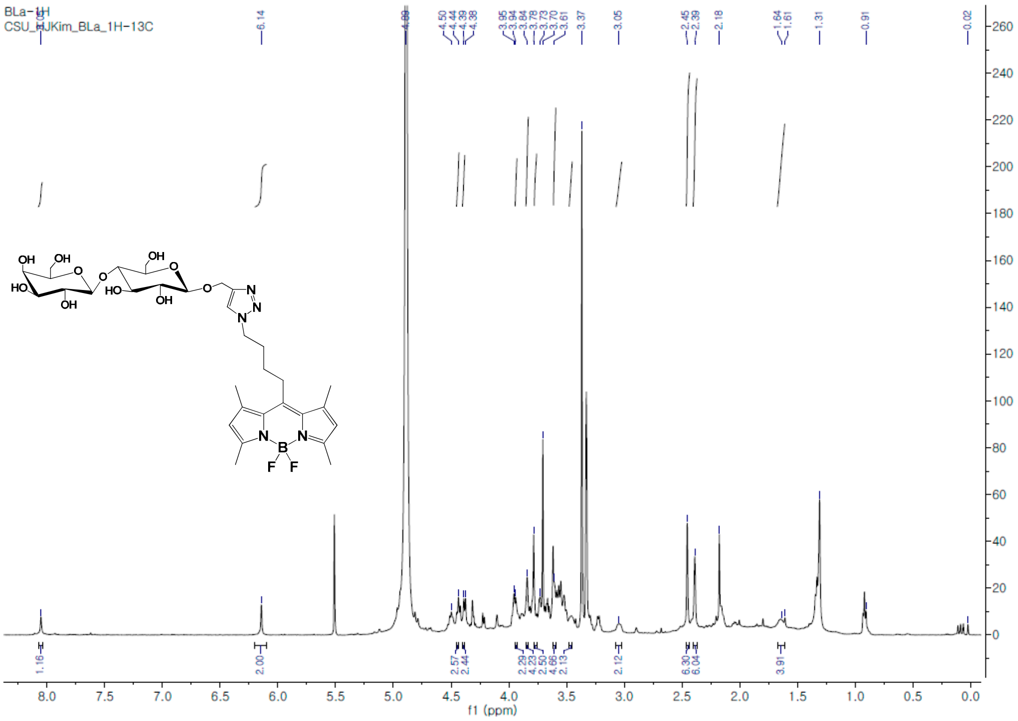

Figure S14. ^{1}H-NMR spectrum of tumor-targeted water-soluble BODIPY BLa.

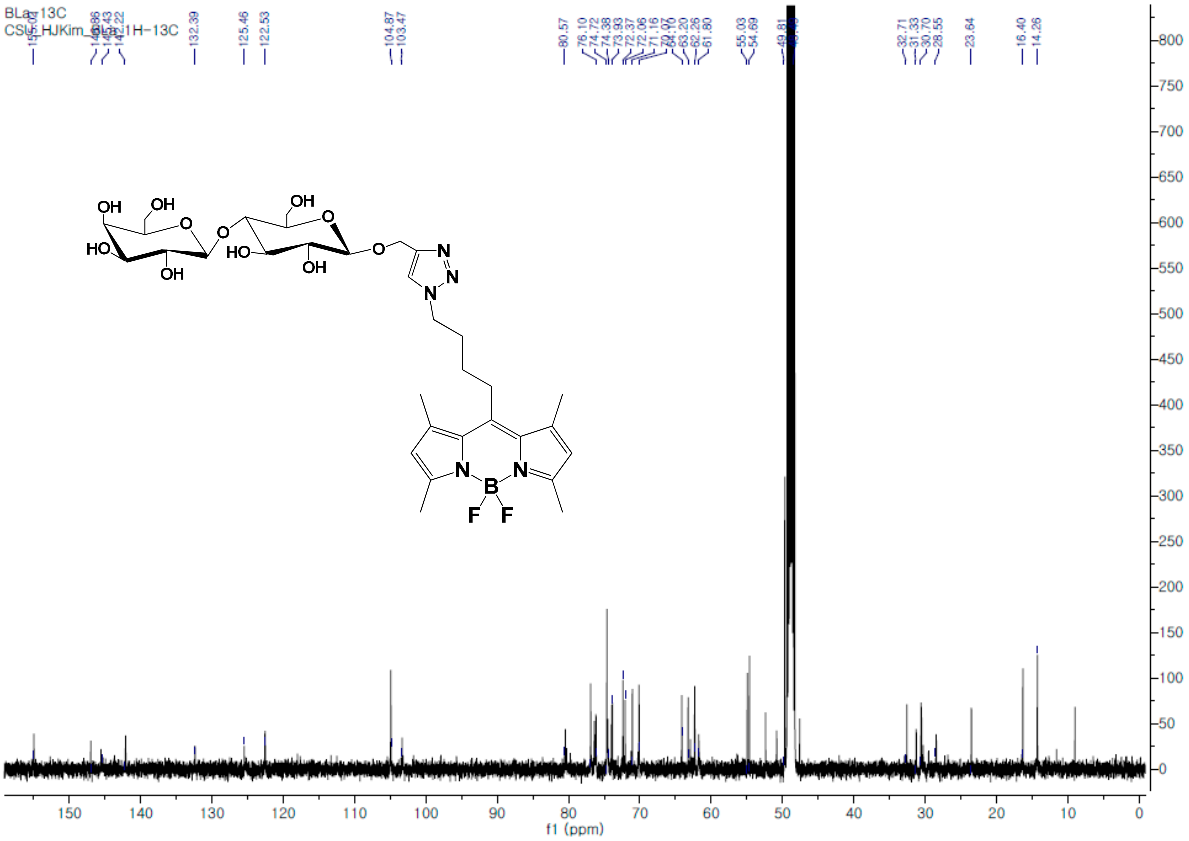

Figure S15. ^{13}C-NMR spectrum of tumor-targeted water-soluble BODIPY BLa.

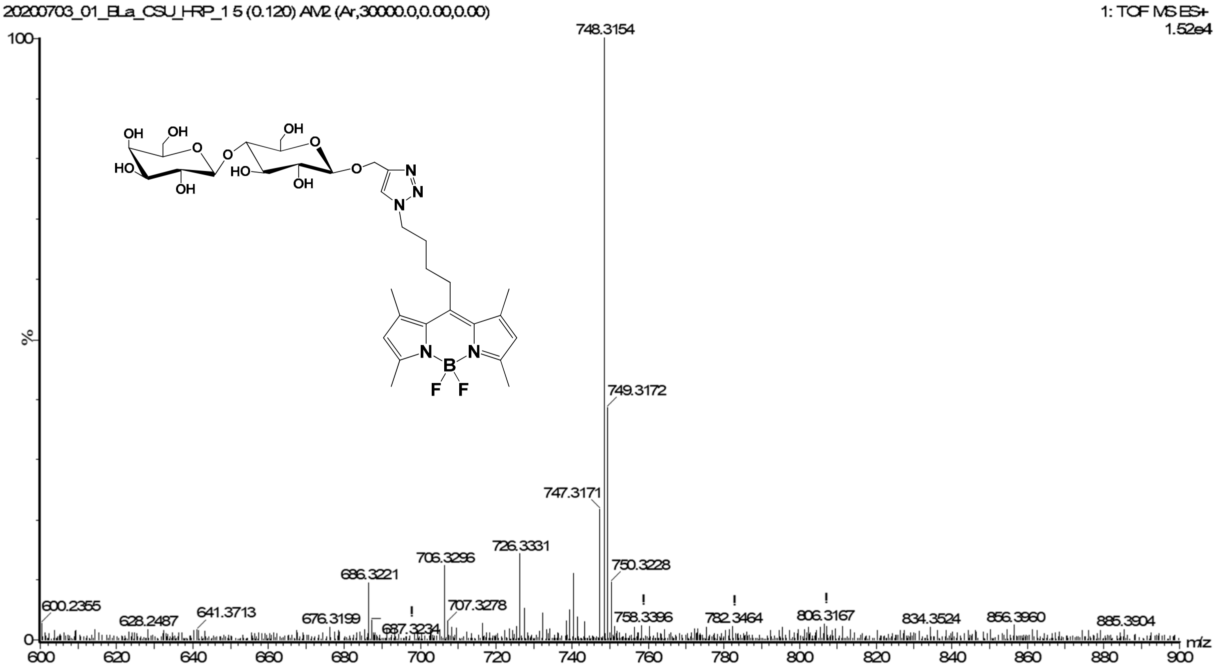

Figure S16. HR-ESI mass spectrum of tumor-targeted water-soluble BODIPY BLa.

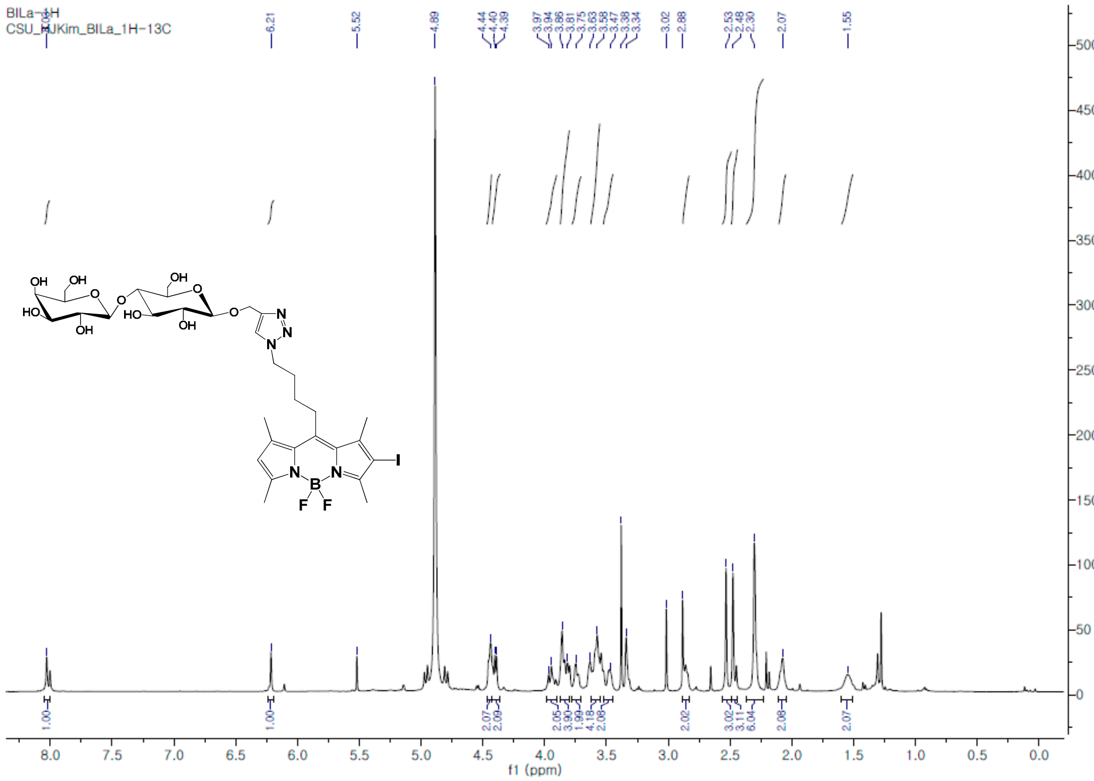

Figure S17. ^{1}H-NMR spectrum of tumor-targeted water-soluble BODIPY BILa.

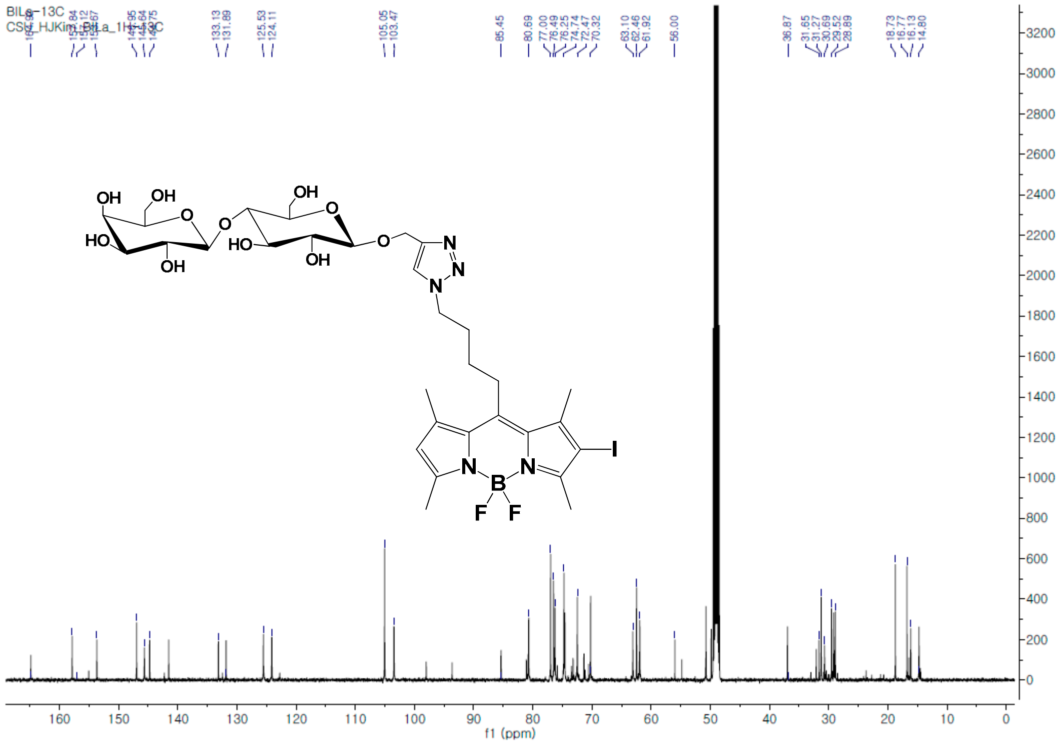

Figure S18. ^{13}C-NMR spectrum of tumor-targeted water-soluble BODIPY BILa.

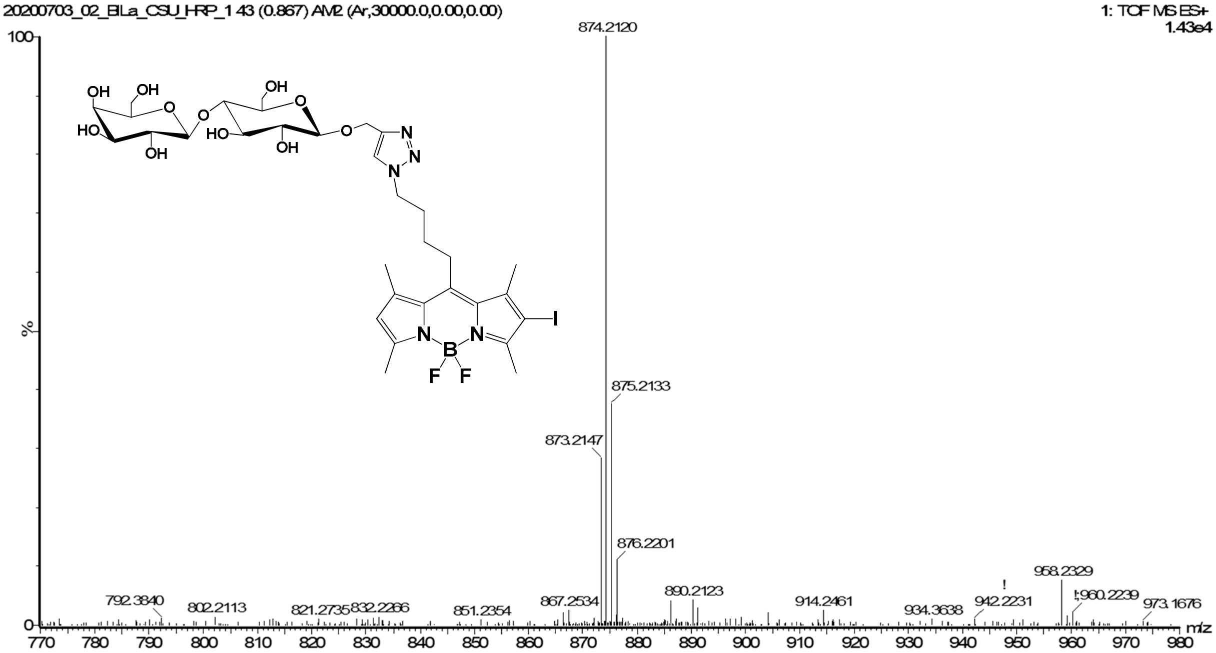

Figure S19. HR-ESI mass spectrum of tumor-targeted water-soluble BODIPY BILa.

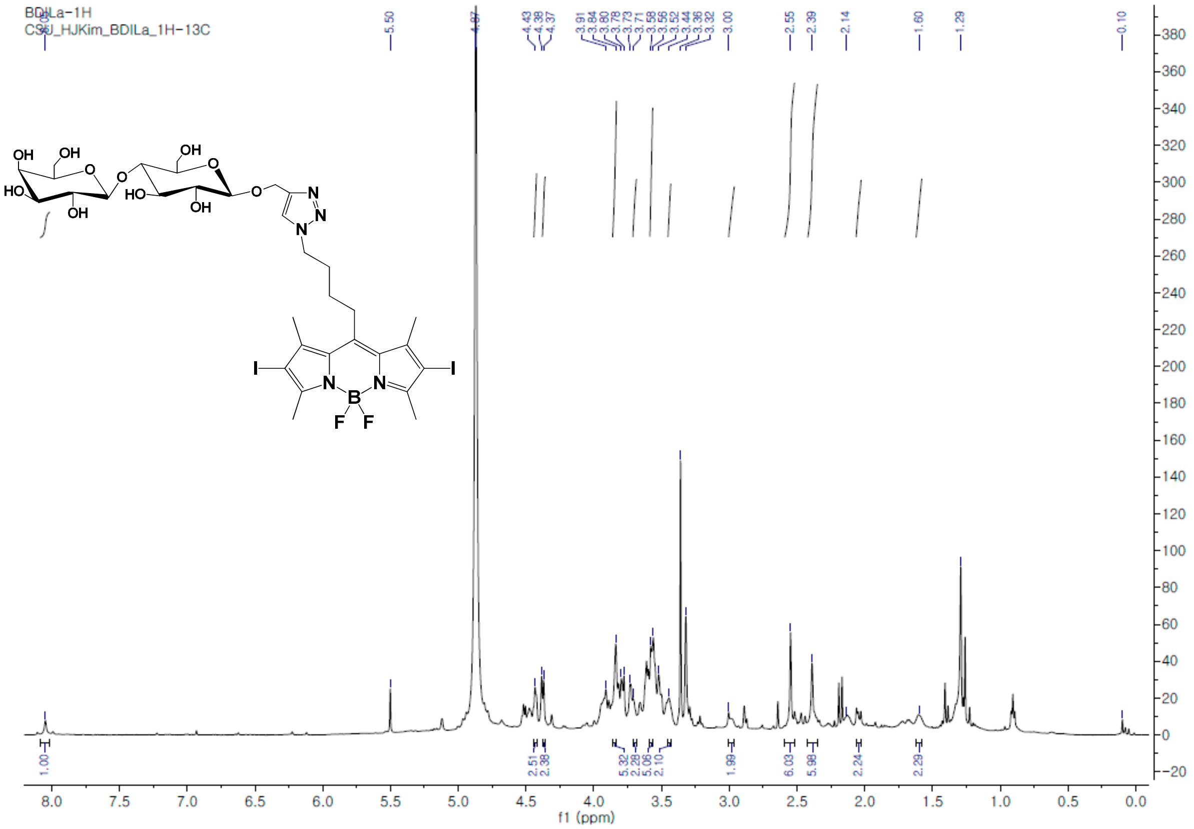

Figure S20. ^{1}H-NMR spectrum of tumor-targeted water-soluble BODIPY BDILa.

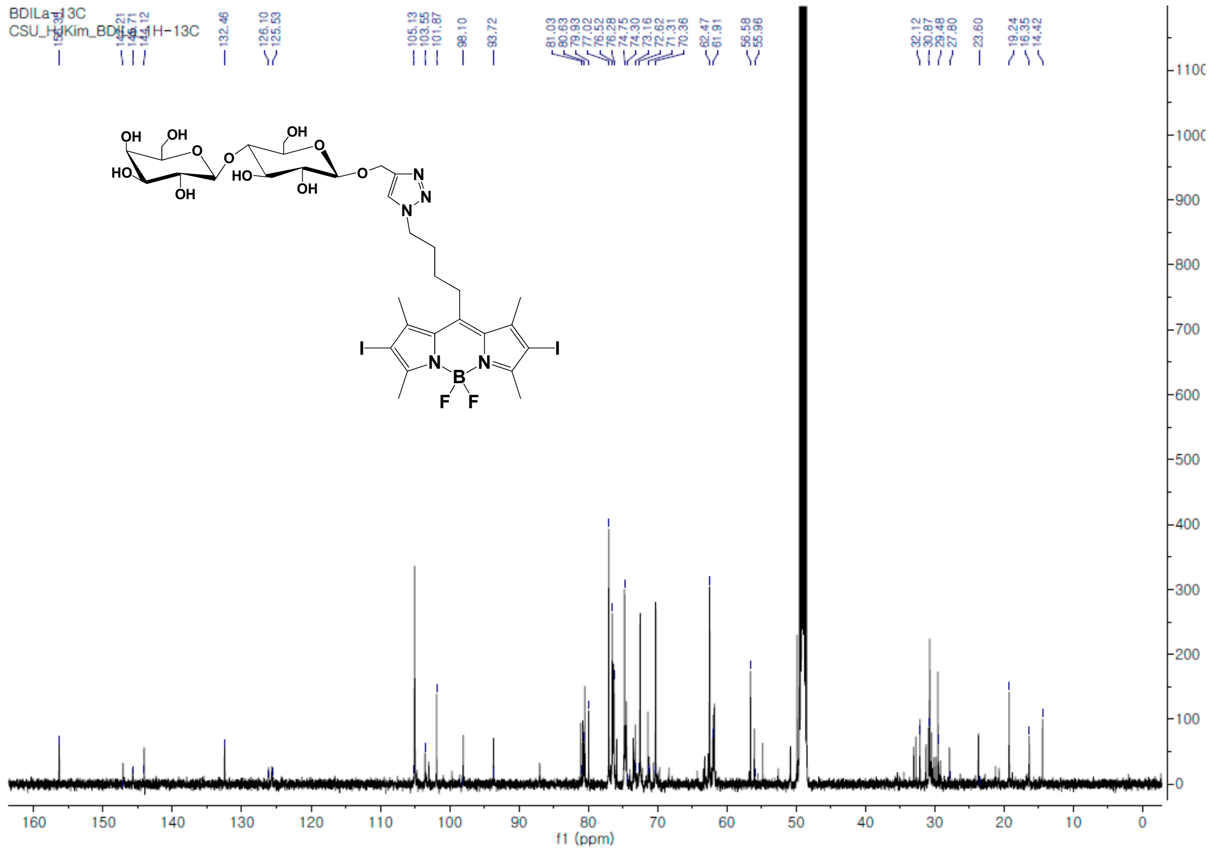

Figure S21. ^{13}C-NMR spectrum of tumor-targeted water-soluble BODIPY BDILa.

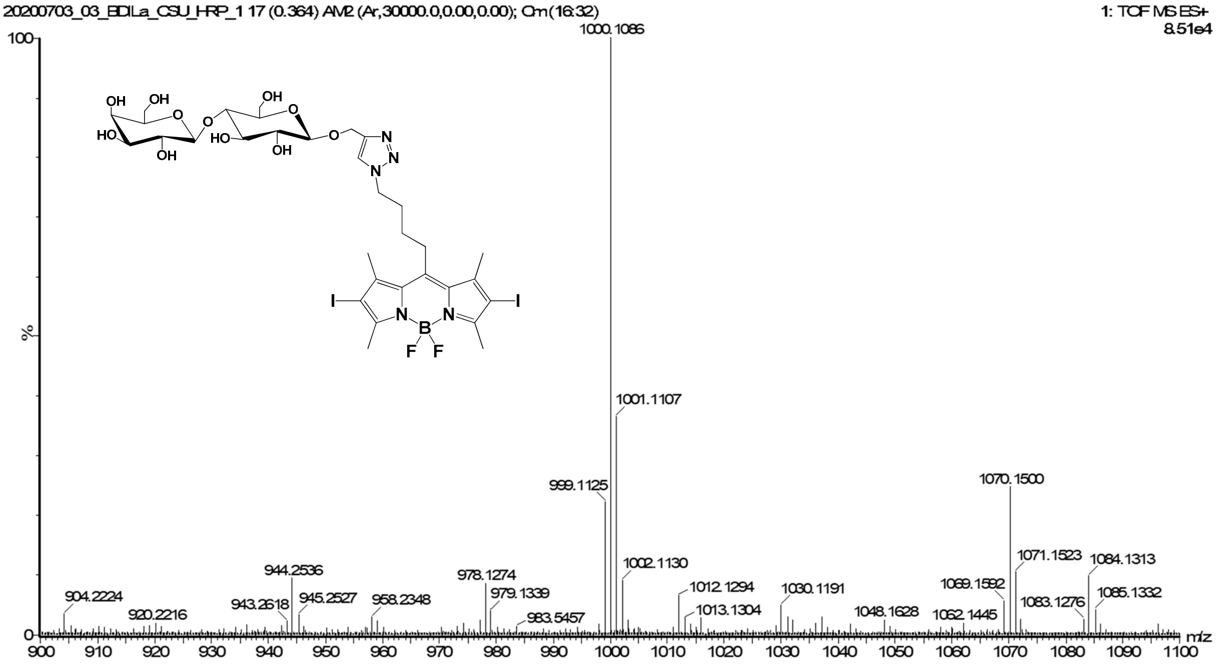

Figure S22. HR-ESI mass spectrum of tumor-targeted water-soluble BODIPY BDILa.

References

1. Geng, J.; Lindqvist, J.; Mantovani, G.; Chen, G.; Sayers, C.T.; Clarkson, G.J.; Haddleton, D.M. Well-Defined Poly (N-glycosyl 1, 2, 3-triazole) Multivalent Ligands: Design, Synthesis and Lectin Binding Studies. *Qsar & Combinatorial Science*, **2007**, *26*, 1220-1228.
2. Matsuo, I.; Isomura, M.; Miyazaki, T.; Sakakibara, T.; Ajisaka, K. Chemoenzymatic synthesis of the branched oligosaccharides which correspond to the core structures of N-linked sugar chains. *Carbohydrate research*, **1997**, *305*, 401-413.
3. van der Peet, P.; Gannon, C.T.; Walker, I.; Dinev, Z.; Angelin, M.; Tam, S.; Ralton, J.E.; McConville, M.J.; Williams, S.J. Use of Click Chemistry to Define the Substrate Specificity of Leishmania β-1, 2-Mannosyltransferases. *ChemBioChem*, **2006**, *7*, 1384-1391.
